# 8-Hydroxyquinoline-5-Sulfonic Acid-Containing Poly (Vinyl Alcohol)/Chitosan Electrospun Materials and Their Cu^2+^ and Fe^3+^ Complexes: Preparation, Antibacterial, Antifungal and Antitumor Activities

**DOI:** 10.3390/polym13162690

**Published:** 2021-08-12

**Authors:** Milena Ignatova, Nevena Manolova, Iliya Rashkov, Nadya Markova, Rositsa Kukeva, Radostina Stoyanova, Ani Georgieva, Reneta Toshkova

**Affiliations:** 1 Laboratory of Bioactive Polymers, Institute of Polymers, Bulgarian Academy of Sciences, Acad. G. Bonchev St. Bl. 103A, BG-1113 Sofia, Bulgaria; rashkov@polymer.bas.bg; 2 Institute of Microbiology, Bulgarian Academy of Sciences, Acad. G. Bonchev Bl. 26, BG-1113 Sofia, Bulgaria; markn@bas.bg; 3 Institute of General and Inorganic Chemistry, Bulgarian Academy of Sciences, Acad. G. Bonchev St. Bl. 11, BG-1113 Sofia, Bulgaria; kukeva@svr.igic.bas.bg (R.K.); radstoy@svr.igic.bas.bg (R.S.); 4 Institute of Experimental Morphology, Pathology and Anthropology with Museum, Bulgarian Academy of Sciences, Acad. G. Bonchev St. Bl. 25, BG-1113 Sofia, Bulgaria; ageorgieva@bas.bg (A.G.); rtoshkova@bas.bg (R.T.)

**Keywords:** chitosan, poly(vinyl alcohol), 8-hydroxyquinoline-5-sulfonic acid, electrospinning, antibacterial activity, antifungal activity, antitumor efficacy

## Abstract

Novel poly(vinyl alcohol) (PVA)/chitosan (Ch)-based fibrous materials containing an ionizable model drug, 8-hydroxyquinoline-5-sulfonic acid (SQ), were successfully fabricated by electrospinning. Complexes between the components of the crosslinked PVA/Ch/SQ mats and Cu^2+^ and Fe^3+^ ions were formed. The coordination of these ions in the mats was examined by electron paramagnetic resonance spectroscopy (EPR). The microbiological screening against *S. aureus* and *C. albicans* revealed that both the incorporation of SQ in the mats and the complexation with Cu^2+^ and Fe^3+^ imparted to these materials antibacterial and antifungal activities. Moreover, the SQ-containing mats and their complexes displayed good cytotoxicity against human cervical HeLa tumor cells. The most prominent was the cytotoxicity of the Cu^2+^ complex of the mats. The combined antibacterial, antifungal and in vitro antitumor activities render these novel materials promising candidates for wound dressing applications and for application in the local treatment of cervical tumors.

## 1. Introduction

Electrospinning is a cutting-edge technology for producing continuous polymer fibers with diameters ranging from a few nanometers to several micrometers [1,2]. One of the most promising applications of electrospun materials is in the biomedical field. During the last decade, electrospun micro- and nanofibrous materials have been considered as carriers of low-molecular-weight compounds with antimicrobial and antitumor properties [3,4]. This is due to some important characteristics of the fibrous materials, such as their high specific surface area and highly porous structure [5]. These properties contribute to the attainment of sustained and controlled drug release and are a prerequisite for improving the therapeutic effect of drugs and diminishing their undesirable side effects [6,7,8]. Electrospun fibrous carriers offer possibilities for site-specific drug delivery [9,10]. Moreover, high loading capacity and high encapsulation efficiency of the incorporated drugs can be achieved by electrospinning [11,12]. Chitosan (Ch), one of the most widely used natural polymers [13,14], is of interest as a drug carrier due to its complex of beneficial properties such as non-toxicity, biodegradability, biocompatibility and the presence of functional reactive groups [15]. In addition, Ch possesses good antimicrobial, antifungal and antitumor properties [16,17,18]. However, the formation of defect-free, continuous Ch-based fibers by electrospinning encounters some difficulties due to its polyelectrolyte nature. For the first time, Ch-containing fibers were successfully obtained by electrospinning of Ch in the presence of a non-ionogenic polymer, such as poly(ethylene oxide) [19,20] or poly(vinyl alcohol) (PVA) [21], or by electrospinning of pure Ch using trifluoroacetic acid as a solvent [22,23]. PVA was selected for incorporation into fibrous materials intended for biomedical purposes since this polymer is biocompatible and has low toxicity and functional reactive groups. To date, there are a limited number of reports on the preparation of electrospun materials from mixed PVA/Ch containing drugs such as antibiotics and antitumor drugs [24,25,26,27,28,29] or biologically active compounds of natural origin (curcumin) [30].

Recently, aromatic heterocyclic compounds from the group of 8-hydroxyquinoline, which have useful properties—antibacterial, antifungal, antitumor, antioxidant, anti-inflammatory and antiviral—and are characterized by low toxicity [31,32,33,34], have been studied intensively. Previously, we have reported on the incorporation of 8-hydroxyquinoline derivatives into electrospun materials from synthetic polymers [35,36,37], chitosan and its derivatives [19,38] and other natural polymers [39,40]. We have also reported the chemical immobilization of 5-amino-8-hydroxyquinoline onto the surface of electrospun fibers from styrene/maleic anhydride copolymers [41]. Recently, we have obtained fibrous materials by electrospinning of polylactide and Jeffamine ED^®^ containing covalently bound 8-hydroxyquinoline-2-carboxaldehyde or its Cu^2+^ complex [42].

In the present study, 8-hydroxyquinoline-5-sulfonic acid (SQ) was selected as a model compound of the 8-hydroxyquinoline group. It has been reported that SQ exerts good antibacterial, antifungal and antitumor activity [43,44,45]. The incorporation of this biologically active compound into electrospun fibrous materials can impart advantageous biological properties to the materials. The presence of an ionizable sulfo group in SQ may allow ionic interaction between the SQ and the Ch molecules of the Ch-containing fibers. Moreover, the nitrogen atom of the ring as well as the hydroxyl group remain available for complexation with transition metal ions (Cu^2+^, Fe^2+^, Fe^3+^, etc.) of biological significance. The chelating ability of 8-hydroxyquinolines has been proposed to account for their biological activity [31,32]. It has been demonstrated that the biological activity of 8-hydroxyquinolines was enhanced by their complexation with Cu or Fe ions [46,47]. Thus, in the present study, Cu^2+^ and Fe^3+^ ions have been selected for coordination with SQ. Furthermore, to the best of our knowledge, there are no data on the preparation of fibrous materials from PVA and Ch, in which SQ is incorporated.

The present contribution aims at studying the possibility of obtaining novel SQ-containing materials from PVA/Ch by electrospinning. The coordination of the ions in Cu^2+^ (Fe^3+^) complexes of PVA/Ch/SQ mats was examined. The morphology, the composition and the thermal characteristics of the fibrous materials were studied. The antibacterial and antifungal activity of the SQ-containing mats and their complexes against Gram-positive bacteria *S. aureus* and fungi *C. albicans* was evaluated. The impact of the composition of the obtained fibrous mats on their in vitro antitumor efficacy against human cervical HeLa tumor cell lines was also monitored.

## 2. Materials and Methods

### 2.1. Materials

Poly(vinyl alcohol) (PVA) (96% hydrolyzed, Mw approx. 85,000–124,000 g/mol, Acros Organics, Geel, Belgium), 8-hydroxyquinoline-5-sulfonic acid (SQ) (Aldrich, St. Louis, MO, USA), anhydrous CuCl_2_ (Acros Organics, Geel, Belgium) and anhydrous FeCl_3_ (Acros Organics, Geel, Belgium) were of analytical-grade purity and were used as received. Chitosan (Ch) with an average viscometric molar mass of 380,000 g/mol (Aldrich, St. Louis, MO, USA) and a deacetylation degree of 80% was used. Glutaraldehyde (50% *v*/*v*), glacial acetic acid, absolute ethanol and all salts used for the preparation of buffer solutions (KH_2_PO_4_, Na_2_HPO_4_, NaOH), were purchased from Merck Chemicals (Merck, Billerica, MA, USA). The buffer solutions of pH 4.5 (CH_3_COOH/NaOH) and pH 7.4 (KH_2_PO_4_/Na_2_HPO_4_) were used. The concentration of the reagents was chosen in such a way as to achieve a value of the ionic strength equal to 0.1.

HeLa human cervical cancer cells (ATCC, CCL-2) were obtained from the American Type Cultures Collection (ATCC, Rockville, MD, USA). Acridine orange (AO), ethidium bromide (EtBr), and 3-(4,5-dimethylthiazol-2-yl)-2,5-diphenyltetrazolium bromide (MTT) were purchased from Sigma-Aldrich, Schnelldorf, Germany. Meanwhile, 4′,6-diamidino-2-phenylindole (DAPI) was supplied by AppliChem, Darmstadt, Germany. All culture reagents, namely Dulbecco’s Modified Eagle’s Medium (DMEM) (Sigma-Aldrich, Schnelldorf, Germany), fetal bovine serum (FBS) (Gibso/BRL, Grand Island, NY, USA), glutamine, penicillin and streptomycin (LONZA, Cologne, Germany), were used as received. The disposable consumables were supplied by Orange Scientific, Braine-l’Alleud, Belgium. *Staphylococcus aureus* (*S. aureus*) 3359 and *Candida albicans* (*C. albicans*) 74 were purchased from the National Bank for Industrial Microorganisms and Cell Cultures (NBIMCC), Sofia, Bulgaria.

### 2.2. Preparation of the Fibrous Materials

#### 2.2.1. Preparation of PVA/Ch Mats

First, 2 wt% Ch solution was prepared by dissolving 0.2 g Ch in 10 mL diluted acetic acid (1% *v*/*v*) under continuous stirring for 24 h at room temperature. Then, 2.0 g PVA was placed in 20 mL deionized water at 80 °C to obtain a homogenous 10 wt% PVA solution by stirring for 12 h. The spinning solutions PVA/Ch were prepared by mixing PVA and Ch solutions at weight ratios of PVA/Ch = 9:1, 8:2 and 7:3 under continuous stirring for 4–5 h.

The mixed solutions were placed into a plastic syringe (5 mL) equipped with a needle (gauge: 20GX11/2″). The positive lead of a direct-current custom-made high-voltage power supply was connected to the needle via alligator clips. The electrospinning solutions were delivered using an infusion pump (NE-300 Just InfusionTM Syringe Pump, New Era Pump Systems Inc., Farmingdale, NY, USA) at a controlled flow rate (0.5 mL/h), at a constant value of the applied voltage (20 kV) and constant tip-to-collector distance (15 cm). The electrospun mats were collected on aluminum foil fixed on the rotating grounded drum (of diameter 56 mm) at a rotating speed of 1200 rpm. The electrospun fibrous mats were placed under reduced pressure overnight at 45 °C to remove any solvent residues.

Prior to electrospinning, the dynamic viscosity of the spinning solutions was measured using a Bookfield DV-II+ programmable viscometer (Middleboro, MA, USA) for cone/plate option equipped with a sample thermostat cup and a cone spindle, at 25 ± 0.1 °C. The electrical resistance of the spinning solutions was measured in an electrolytic cell equipped with rectangular sheet platinum electrodes as previously described [48].

#### 2.2.2. Preparation of PVA/Ch/SQ Mats

To prepare PVA/Ch/SQ mats, 0.126 g SQ was added to the 6 g 2 wt% Ch solution in diluted acetic acid (1% *v*/*v*) (molar ratio of (SQ)/(aminoglucoside units of Ch) = 1/1.4). The mixture was stirred for 5 h at room temperature. The spinning solution PVA/Ch/SQ (with PVA/Ch = 8/2 *w*/*w* and SQ content 5 wt% with respect to the polymer weight) was prepared by mixing the above-obtained solution and 24 g 10 wt% PVA aqueous solution under stirring for 7 h at room temperature. The spinning solution PVA/Ch/SQ with PVA/Ch = 8/2 *w*/*w* and SQ content 10 wt% with respect to the polymer weight (molar ratio of (SQ)/(aminoglucoside units of Ch) = 1/0.7) was also prepared using the same procedure. The electrospinning set-up, the nozzle tip/collector distance, the applied voltage and the flow rate for PVA/Ch/SQ were the same as for PVA/Ch spinning solutions.

### 2.3. Crosslinking of the PVA/Ch and PVA/Ch/SQ Mats

In order to stabilize the obtained PVA/Ch and PVA/Ch/SQ mats against dissolving in water, they were crosslinked with glutaraldehyde vapors for 24 h at room temperature. The crosslinked mats (further denoted as cr(PVA/Ch) and cr(PVA/Ch)/SQ) were vacuum-dried at 30 °C.

### 2.4. Preparation of Cu^2+^(Fe^3+^) Complexes of cr(PVA/Ch)/SQ Mats and of SQ

The Cu^2+^(Fe^3+^) complexes of cr(PVA/Ch)/SQ mats were prepared by soaking the mats in 0.1 M CuCl_2_ (FeCl_3_) solution in absolute ethanol at room temperature for 1 h, then repeatedly rinsing with ethanol to remove any non-coordinated salt and drying under reduced pressure. For the sake of comparison, Cu^2+^(Fe^3+^) complexes of crPVA and cr(PVA/Ch) mats were also prepared. These complexes were obtained using the same procedure.

The complex with a molar ratio of SQ and Cu^2+^ 2:1 (further denoted as SQ.Cu^2+^) was prepared by the procedure described in detail elsewhere [49] (for preparation of SQ.Cu^2+^, see Supplementary Material). The complex with a molar ratio of SQ and Fe^3+^ 3:1 (further denoted as SQ.Fe^3+^) was synthesized according to a known procedure [50] (for preparation of SQ.Fe^3+^, see Supplementary Material).

### 2.5. Characterization

The morphology of the mats was studied by SEM. The samples were vacuum-coated with gold and examined by a Jeol JSM-5510 SEM (Tokyo, Japan). The fiber morphology was assessed using the ImageJ software (V.1.53e, Wayne Rasband, National Institute of Health, Bethesda, MD, USA) by measuring at least 30 fibers from each SEM image.

Attenuated total reflection Fourier-transform infrared (ATR-FTIR) spectra were registered using an IRAffinity-1 spectrophotometer (Shimadzu Co., Kyoto, Japan) equipped with a MIRacle^TM^ ATR (diamond crystal; depth of penetration of the IR beam into the sample was approximately 2 µm) accessory (PIKE Technologies, Madison, WI, USA). The surface chemical composition of the fibrous materials was determined by XPS. The XPS measurements were performed in the ultrahigh-vacuum (UHV) chamber of an ESCALAB-MkII (VG Scientific) spectrometer using Mg Kα excitation with a total resolution of ca. 1 eV. Energy calibration was carried out taking the C1s line at 285 eV as a reference.

The differential scanning calorimetric (DSC) studies were performed using a DSC TA Instruments (DSC Q2000, New Castle, DE, USA) apparatus in the temperature range from 20 to 400 °C at a heating rate of 10 °C/min in nitrogen atmosphere.

The static water contact angles of the mats were measured using an Easy Drop Krüss GmbH apparatus (DSA 10-MK2 model, Hamburg, Germany) at room temperature. The mats were cut at 0° and at 90° with respect to the collector rotation direction. A drop of deionized water (10 µL) was deposited onto the mats (cut in the collector rotation direction). From the images of the droplets on the surface of the mats acquired by a digital camera and processed by a software program, the average values of the water contact angles were determined based on at least 20 measurements for each sample.

The EPR spectra of Fe^3+^ and Cu^2+^ complexes of crPVA, cr(PVA/Ch), cr(PVA/Ch)/SQ mats were recorded as the first derivative of the absorption signal by using a Bruker EMXplus EPR spectrometer (E7001039, Karlsruhe, Germany), operating in the X-band (9.4 GHz). The recording temperature was varied within the range of −173.15 °C to 21.85 °C. The EPR spectra were simulated through the program SIMFONIA (Bruker). For all measurements, a quartz tube, finger dewar and flat cell were used. The manipulation of the complexes was performed in a glove-box containing less than 5 ppm O_2_ and H_2_O.

In order to determine the stability of cr(PVA/Ch) and cr(PVA/Ch)/SQ mats in acetate buffer of pH 4.5 (CH_3_COONa/CH_3_COOH), the mats were immersed in the buffer for 24 h. The treated samples were repeatedly rinsed with distilled water, vacuum-dried and the fiber morphology was analyzed by a Jeol JSM-5510 SEM (Tokyo, Japan) microscope. To determine the weight losses of cr(PVA/Ch) and cr(PVA/Ch)/SQ mats in acetate buffer of pH 4.5, the mats were immersed in acetate buffer for 24 h. 

The swelling degree (α) of cr(PVA/Ch) and cr(PVA/Ch)/SQ mats after 24 h in acetate buffer of pH 4.5 was determined gravimetrically and was calculated from Equation (1):

α% = (weight of swollen mats − weight of dry mats)/weight of dry mats × 100(1)

### 2.6. In Vitro SQ, SQ.Cu^2+^ and SQ.Fe^3+^ Release

A sample of SQ-containing fibrous mats and their Cu^2+^(Fe^3+^) complexes (16 mg) was placed in a vial filled with 100 mL of phosphate-buffered saline (PBS) (KH_2_PO_4_/Na_2_HPO_4_, pH 7.4 and ionic strength of 0.1) containing Tween 40 (PBS/Tween 40 = 99/1 *v*/*v*) stirred at 100 rpm and incubated at 37 °C in a thermally controlled shaking water bath (JULABO SW23, Allentown, PA, USA). Aliquots were withdrawn at predetermined time intervals and their absorbance was recorded by a DU 800 UV–vis spectrophotometer (Beckman Coulter, CA, USA) at a wavelength of 305 nm for SQ-containing mats, of 368 nm for SQ.Cu^2+^-containing mats and of 356 nm for SQ.Fe^3+^-containing mats. The amount of released SQ, SQ.Cu^2+^(Fe^3+^) was calculated using calibration curves (correlation coefficient R = 0.999) for the mats in PBS/Tween 40 (99/1 *v*/*v*), pH = 7.4, ionic strength 0.1. All SQ, SQ.Cu^2+^ and SQ.Fe^3+^ release tests were performed in triplicate. The mechanism of SQ, SQ.Cu^2+^(Fe^3+^) release was studied by using the Korsmeyer–Peppas model [51]:

M_t_/M_∞_ = Kt^n^ (2)

where M_t_ is the amount of drug released at time t, M_∞_ is the total amount of drug incorporated in the mats, K is the kinetic constant, and n is the release exponent. 

### 2.7. Microbiological Tests

The minimum inhibitory concentration (MIC) of SQ and its Cu^2+^ and Fe^3+^ complexes was determined for Gram-positive bacteria *S.aureus* 3359 (NBIMCC, Sofia, Bulgaria) and for fungi *C. albicans* 74 (NBIMCC, Sofia, Bulgaria), respectively (for determination of MIC, see Supplementary Material).

The antibacterial and antifungal activity of crPVA, cr(PVA/Ch), cr(PVA/Ch)/SQ and Fe^3+^(Cu^2+^) complex of cr(PVA/Ch)/SQ mats against Gram-positive bacteria (*S. aureus* 3359) and fungi (*C. albicans* 74) was tested by the disk diffusion assay. The tests were performed using Tryptic Soy Agar (TSA, Becton Dickinson, Heidelberg, Germany) solid medium for *S.aureus* and Sabouraud Dextrose Agar (SDA, Becton Dickinson, Sparks, MD, USA) solid medium for *C. albicans*. The surface of standard Petri dishes with solid agar was inoculated with a suspension of 24-h-cultured *S. aureus* or 48-h-cultured *C. albicans*, respectively, at a concentration of 1 × 10^5^ cells/mL. Within 5–10 min after inoculation, each sample was placed on the inoculated surface (one disk with a diameter of 13 mm and weight 4.0 mg per Petri dish). The Petri dishes with *S. aureus* were incubated for 24 h at 37 °C and those with *C. albicans* for 48 h at 37 °C. Subsequently, the average diameters of the zones of inhibition around the disks were determined using the ImageJ software based on 10 measurements in 10 different directions for each zone. 

Evaluation of the interaction between *S. aureus* 3359 and crPVA, cr(PVA/Ch), cr(PVA/Ch)/SQ and Fe^3+^(Cu^2+^) complex of cr(PVA/Ch)/SQ mats was performed by SEM observation with Jeol JSM-5510 (Jeol Ltd., Tokyo, Japan) of *S. aureus* cells adhered to the mat surface that had been in contact with a bacteria suspension. Briefly, the mats were incubated in 2.0 mL of culture of *S. aureus* (containing ca. 10^7^ cells/mL) at 37 °C for 24 h. Then, the samples were first washed twice with phosphate-buffered saline (PBS, pH 7.4) for the removal of non-adhered bacteria. The adhered bacteria on the mat surface were fixed by immersion of the mats in 2.0 mL of 2.5 vol% glutaraldehyde solution in PBS at 4 °C for 5 h. The samples were washed carefully with PBS again and freeze-dried. The morphology of the *S. aureus* cells after contact with the mats was observed by an SEM Jeol JSM-5510 (Tokyo, Japan) after vacuum gold-coating (Jeol JFC-1200 fine coater).

### 2.8. MTT Cytotoxicity Assay

HeLa cells were cultured in DMEM supplemented with 10% FBS, 100 U/mL penicillin and 0.1 mg/mL streptomycin in a CO_2_ incubator at 37 °C and 5% CO_2_. Cells were trypsinized with 0.25% Trypsin–EDTA and counted using a hemocytometer. The cells were placed in 96-well microtiter plates at a concentration of 1 × 10^5^ cells/mL. The culture medium was replaced after overnight incubation at 37 °C in a humid atmosphere with 5% CO_2_ required for cell attachment. Cells were treated with various formulations of fibrous mats (crPVA, cr(PVA/Ch), cr(PVA/Ch)/SQ, Fe^3+^ complex of cr(PVA/Ch)/SQ and Cu^2+^ complex of cr(PVA/Ch)/SQ) preliminarily sterilized by UV light for 30 min and incubated for 24, 48 and 72 h. All SQ-containing mats and their Fe^3+^ (Cu^2+^) complexes were tested at a concentration of SQ 340 μg/mL of culture medium. The concentration of SQ in SQ, SQ.Fe^3+^ and SQ.Cu^2+^ was 340 μg/mL of culture medium. HeLa cells incubated in culture medium only were used as a negative control and HeLa cells incubated in the presence of SQ and its Fe^3+^ and Cu^2+^ complexes were used as a positive control. Each variant of fibrous mats was assayed by five measurements. After culturing in the presence of mats, the HeLa cells were washed twice with PBS (pH 7.4), after which 100 μL of MTT solution was added to each well and the cells were incubated at 37 °C for 3 h. The supernatants were aspirated and 100 μL of the lysing solution (DMSO:ethanol = 1:1) was added to each well in order to dissolve the obtained formazan. The results from the MTT assay were read using an ELISA plate reader (TECAN, SunriseTM, Grodig/Salzburg, Austria). The percentage of cell viability was calculated as follows:

cell viability (%) = OD_570_ (experimental)/OD_570_ (control) × 100 (3)

### 2.9. Studying Apoptosis by Fluorescent Staining Methods

#### 2.9.1. Double Staining Assay with AO and EtBr

Apoptotic nuclear morphology was assessed using AO and EtBr double staining similarly to standard procedure [52]. In brief, HeLa cells (1 × 10^5^ cells/mL) seeded on glass lamellas (12 mm Ø) placed in 24-well plates were incubated overnight at 37 °C to allow the cells to adhere and subsequently were cultured in the presence of various fibrous mats (crPVA, cr(PVA/Ch), cr(PVA/Ch)/SQ, Fe^3+^ complex of cr(PVA/Ch)/SQ and Cu^2+^ complex of cr(PVA/Ch)/SQ) for 24 h. Untreated HeLa cells were used as a negative control and HeLa cells treated with SQ and its Fe^3+^ and Cu^2+^ complexes were used as a positive control. After incubation, the mats were removed and glass lamellas were washed twice with PBS (pH 7.4) to remove unattached cells. The cells were then stained with fluorescent dyes—AO and EtBr—at a ratio of 1:1 (10 µg/mL), mounted onto glass slides and examined immediately under a fluorescence microscope (Leica DM 5000 B, Wetzlar, Germany).

#### 2.9.2. DAPI Staining

The DAPI staining was performed as previously described [53]. HeLa cells (1 × 10^5^ cells/well) were cultivated on glass cover slips in 24-well plates, in a CO_2_ incubator, for 24 h and then exposed to the fibrous mats (crPVA, cr(PVA/Ch), cr(PVA/Ch)/SQ, Fe^3+^ complex of cr(PVA/Ch)/SQ and Cu^2+^ complex of cr(PVA/Ch)/SQ). After 24 h, the cells were rinsed with PBS (pH 7.4) and fixed with methanol at room temperature and subsequently stained with a DAPI solution for cell nuclei observation by a fluorescence microscope (Leica DM 5000 B, Wetzlar, Germany).

### 2.10. Statistical Analysis 

The data were displayed as means ± standard deviation (SD). The statistical significance of the results was assessed by one-way analysis of variance (ANOVA), followed by post hoc comparison test (Bonferroni) with the use of GraphPAD PRISM software, version 5 (GraphPad Software Inc., San Diego, CA, USA). Values of * *p* < 0.05, ** *p* < 0.01 and *** *p* < 0.001 were considered statistically significant. 

## 3. Results and Discussion

### 3.1. Morphology of the Fibrous Materials

**Figure 1:**
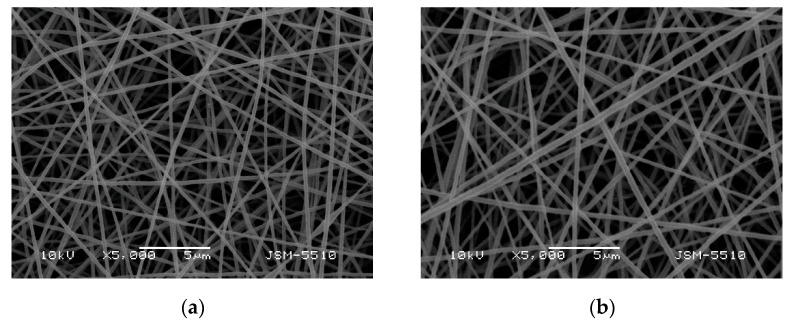
SEM micrographs of electrospun mats of: PVA/Ch/SQ (5 wt% SQ) (**a**), PVA/Ch/SQ (10 wt% SQ) (**b**); magnification × 5000.

Previously, it has been demonstrated that the formation of fibrous materials from ionogenic polymers by electrospinning could be favored by the presence of a non-ionogenic polymer with a flexible chain in the spinning solution [15,19,20,21,48]. In the present study, we have selected PVA as a suitable non-ionogenic partner for mixing with Ch, as it has been shown to facilitate its electrospinning [15,21]. Studies were performed to determine the effect of the composition of PVA/Ch spinning solutions on the morphology of the fibers. This allowed us to select such a PVA/Ch weight ratio for the incorporation of SQ in which defect-free PVA/Ch fibers are formed. By varying the PVA/Ch weight ratio, mats with different morphology were formed (Supplementary Material, Figure S1). Defect-free continuous fibers with a cylindrical shape and an average diameter of 147 ± 56 nm were obtained by electrospinning of solutions at a weight ratio PVA/Ch = 8:2 (Supplementary Material, Figure S1c). A further increase in the content of Ch (PVA/Ch = 7:3) resulted in the preparation of fibers with a smaller average diameter (134 ± 53 nm) and in the formation of spindle-like defects (Supplementary Material, Figure S1d). These changes in the morphology might be explained by the increase in the solution’s conductivity on increasing the Ch content (Supplementary Material, Table S1). Therefore, spinning solutions with weight ratio of PVA/Ch = 8:2 were selected for further experiments to incorporate SQ into the PVA/Ch fibers. SQ content was chosen in such a manner that (SQ)/(aminoglucoside units of Ch) was close to 1:1 (mol/mol)—1:1.4 (mol/mol) and 1:0.7 (mol/mol) for SQ content 5 wt% and 10 wt% with respect to the polymer weight, respectively. Continuous, defect-free and cylindrical PVA/Ch/SQ fibers were obtained (Figure 1a,b). The average diameter of these fibers changed insignificantly compared to that of PVA/Ch fibers (Supplementary Material, Figure S1c) to 159 ± 55 nm and 230 ± 68 nm for PVA/Ch mats containing 5 and 10 wt % SQ, respectively. The observed effect might be attributed to the decrease in the conductivity of the spinning solutions containing SQ compared to the PVA/Ch spinning solution (Supplementary Material, Table S1). The viscosity of PVA/Ch solutions remained unchanged on addition of 5 and 10 wt% SQ (Supplementary Material, Table S1).

**Figure 2:**
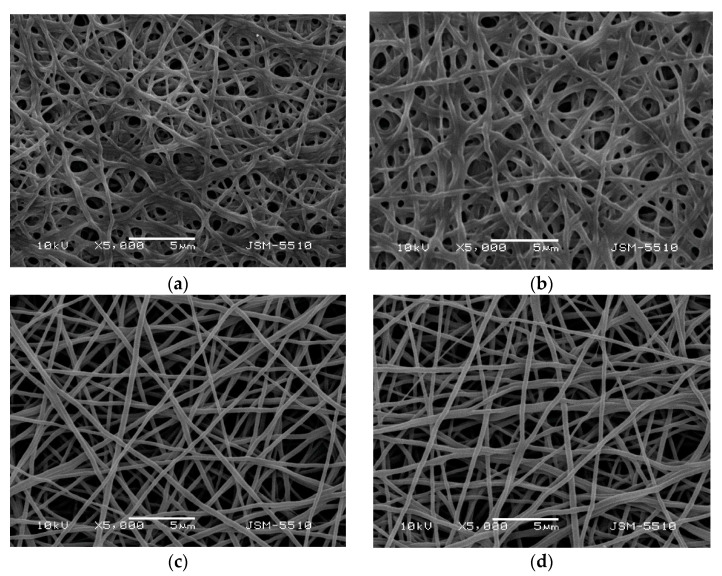
SEM micrographs of electrospun mats of: cr(PVA/Ch) (8:2 *w*/*w*) (**a**) and cr(PVA/Ch)/SQ (10 wt% SQ) (**b**) after 24 h immersion in acetate buffer of pH 4.5; cr(PVA/Ch)/SQ (10 wt% SQ) after immersion in ethanol solution of CuCl_2_ (**c**) and FeCl_3_ (**d**) for 1 h.

In order to impart water insolubility to SQ-containing PVA/Ch mats with a view to their potential biomedical application, covalent crosslinking of fibers with glutaraldehyde vapors was performed. It was found that the crosslinking did not cause any change in the morphology of SQ-(non)containing PVA/Ch mats (SEM micrographs not shown). The stability of crosslinked PVA/Ch and PVA/Ch/SQ fibers was studied in acetate buffer of pH 4.5, a good solvent for Ch. The fibers swelled (Figure 2a,b), but even after 24 h, they did not dissolve when placed in contact with the buffer solution. The average diameter of the crosslinked fibers was found to increase to 258 ± 78 nm for cr(PVA/Ch) mats and to 342 ± 89 nm for cr(PVA/Ch)/SQ mats, respectively. Furthermore, the determined weight loss of the SQ-containing mats (near 9%) was close to the amount of the incorporated SQ. No weight loss was observed for the cr(PVA/Ch) mats after their immersion in buffer of pH 4.5, thus evidencing successful crosslinking. The equilibrium swelling degree (α_eq_) determined in acetate buffer of pH 4.5 at 25 °C, for cr(PVA/Ch)/SQ mats, was 240 ± 7% (Supplementary Material, Figure S2). 

The ability of SQ-containing cr(PVA/Ch) mats to form a complex with Cu^2+^ or Fe^3+^ in ethanol was studied. After washing, the mats became greenish-yellow or dark green in color, which is a color characteristic of Cu^2+^ or Fe^3+^ engaged in complexes with SQ. It was found that the immersion of the cr(PVA/Ch)/SQ mats in ethanol solution of CuCl_2_ and FeCl_3_ for 1 h did not cause any change in the morphology of the fibers (Figure 2c,d). The average diameter of the cr(PVA/Ch)/SQ mats slightly increased after obtaining the Cu^2+^ complex from 230 ± 68 nm to 255 ± 70 nm, and after obtaining the Fe^3+^ complex, it changed from 230 ± 68 nm to 242 ± 74 nm.

### 3.2. ATR-FTIR Analysis of the Fibrous Materials

Non-crosslinked and crosslinked PVA/Ch/SQ mats and their Cu^2+^ and Fe^3+^ complexes were characterized by ATR-FTIR spectroscopy. As seen in Figure S3a, the ATR-FTIR spectrum of the non-crosslinked PVA/Ch mats showed the characteristic bands of the PVA and Ch (for ATR-FTIR characteristic bands, see Supplementary Material). The incorporation of SQ into the cr(PVA/Ch) mats was confirmed by ATR-FTIR spectroscopy (Supplementary Material, Figure S4c). A new band at 1500 cm^−1^ characteristic of the stretching vibrations of the SQ ring [54] (for ATR-FTIR characteristic bands of SQ, see Supplementary Material) was detected. The bands corresponding to SO_2_ stretching vibrations and SOH bending vibrations of the sulfo group of SQ in the spectrum of the cr(PVA/Ch)/SQ mat (Supplementary Material, Figure S4c), which were observed in the SQ spectrum at 1038 cm^−1^ and 1177 cm^−1^ (Supplementary Material, Figure S4a), were shifted to a higher wavenumber by 9 cm^−1^ and by 23 cm^−1^, respectively. In the spectrum of the cr(PVA/Ch)/SQ mat (Supplementary Material, Figure S4c), a band at 1558 cm^−1^ characteristic of the bending N-H deformation of the –NH_3_^+^ group from Ch was observed. The obtained results confirmed that the sulfo group of SQ incorporated into the mat was ionized to the SO_3−_ group, which interacted electrostatically with the protonated amino groups of Ch. The comparison of the ATR-FTIR spectrum of the cr(PVA/Ch)/SQ mats (Supplementary Material, Figure S4c) with that of the non-crosslinked PVA/Ch/SQ mats (Supplementary Material, Figure S4b) showed that some changes were detected (for ATR-FTIR characteristic bands, see Supplementary Material). The observed changes are in accordance with literature data for the crosslinked PVA/Ch system [27]. These findings confirmed the successful crosslinking of PVA and Ch from the PVA/Ch/SQ mat.

The ATR-FTIR spectrum of the cr(PVA/Ch)/SQ mat after immersion in ethanolic solution of Cu^2+^ or Fe^3+^ (Supplementary Material, Figure S4d,e) showed an 8 to 10 cm^−1^ shift of the band for C = N stretching vibration from SQ toward a higher wavenumber to 1599 cm^−1^ and to 1597 cm^−1^ for Cu^2+^ and Fe^3+^ complexes of the cr(PVA/Ch)/SQ mat, respectively, compared to the spectrum of SQ (1589 cm^−1^) (Supplementary Material, Figure S4a). This suggested that the lone pair on the nitrogen is involved in bonding with the metal ion. In addition, the broad band at 3300 cm^−1^, corresponding to the stretching vibrations of the hydroxyl and amino groups, became less intense and sharper than that of the cr(PVA/Ch)/SQ mat (Supplementary Material, Figure S4c), which was an indication of the interaction between these groups and Cu^2+^ or Fe^3+^.

### 3.3. Thermal Characteristics of the Fibrous Materials

**Figure 3:**
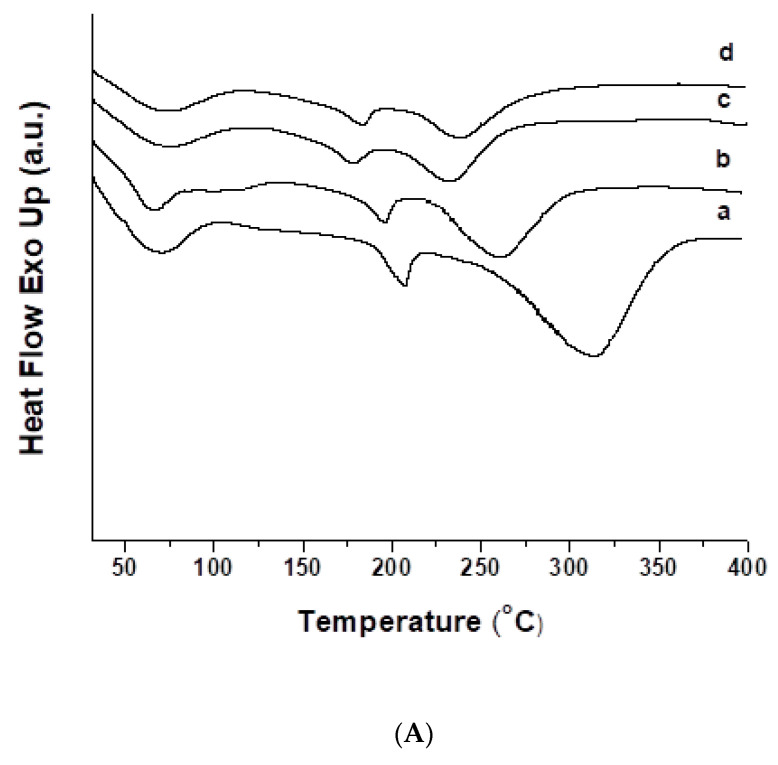

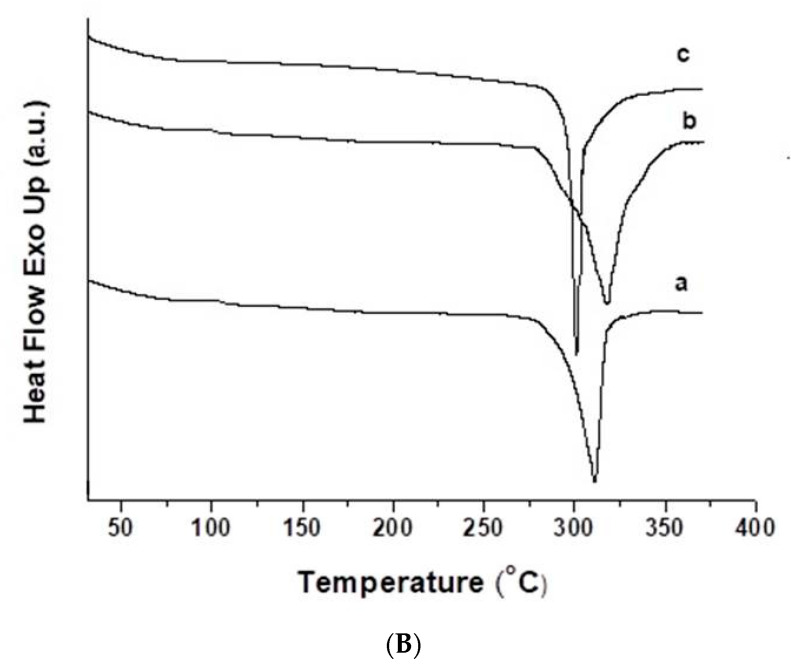
DSC thermograms of fibrous materials from (**A**): cr(PVA/Ch) (a), cr(PVA/Ch)/SQ (10 wt% SQ) (b), Cu^2+^ complex of cr(PVA/Ch)/SQ (10 wt% SQ) (c) and Fe^3+^ complex of cr(PVA/Ch)/SQ (10 wt% SQ) (d). For the sake of comparison, the DSC thermograms from (**B**): SQ (a), SQ.Cu^2+^ (b) and SQ.Fe^3+^ (c) are also presented.

**Figure 4:**
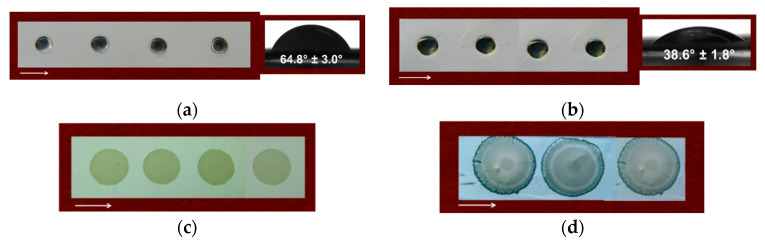
Digital images of water droplets (10 µL) deposited on the surfaces of fibrous mats from (**a**) cr(PVA/Ch), (**b**) cr(PVA/Ch)/SQ, (**c**) Cu^2+^ complex of cr(PVA/Ch)/SQ and (**d**) Fe^3+^ complex of cr(PVA/Ch)/SQ. The direction of the collector rotation is indicated by an arrow.

The thermal behavior of the cr(PVA/Ch)/SQ mats and their Cu^2+^ and Fe^3+^ complexes was evaluated by DSC in the temperature range of 30 to 400 °C (Figure 3). The cr(PVA/Ch) fibers showed a broad endothermic peak that started at room temperature and ended at approximately 104 °C, with a peak maximum at 71 °C. This is attributed to the desorption of water from PVA and Ch. During heating, decomposition of Ch takes place and melting of Ch was not detected [55]. An endothermic peak corresponding to the melting point (T_m_) of PVA at 207 °C was observed, starting at 180 °C and ending at 216 °C. An endothermic peak with a maximum at 208 °C due to the T_m_ of PVA was detected in the thermograms of the crPVA fibers (Supplementary Material, Figure S5). As seen from Figure 4A(a), the degradation of cr(PVA/Ch) fibers begins at temperature above 220 °C with an endothermic peak with a maximum at 313 °C. It can be assumed that the interactions between PVA, Ch and SQ lead to a shift in the T_m_ of PVA to a lower temperature—at 197 °C for the cr(PVA/Ch)/SQ mat (Figure 3A(b)). The observed endothermic peak with a maximum at 259 °C is most likely due to thermal degradation of the cr(PVA/Ch)/SQ fibers. This peak was shifted to a lower temperature compared to the peak detected in the thermograms of the cr(PVA/Ch) mats (Figure 3A(a)). In addition, it is noteworthy that in the case of the cr(PVA/Ch)/SQ mats, no peak corresponding to the melting point of SQ was observed (T_m_ of SQ is 314 °C, Figure 3B(a)). This indicates that the SQ incorporated into the fibers was in the amorphous state. Cu^2+^ and Fe^3+^ complexes of SQ formed in the cr(PVA/Ch)/SQ mats after their immersion in solution of CuCl_2_ or FeCl_3_ were also in the amorphous state (Figure 3A(c,d)), as evidenced by the absence of an endothermic peak for the melting of SQ.Cu^2+^ (T_m_ of SQ.Cu^2+^ is 321 °C, Figure 3B(b)) and of SQ.Fe^3+^ (T_m_ of SQ.Fe^3+^ is 304 °C, Figure 3B(c)). The thermograms of the Cu^2+^ and Fe^3+^ complexes of the mats (Figure 3A(c,d)) showed a shift in the peaks corresponding to PVA melting to lower temperatures—from 208 °C for the crPVA mat (Supplementary Material, Figure S5) to 178 °C and 182 °C for the Cu^2+^ and Fe^3+^ complexes of the cr(PVA/Ch)/SQ mats, respectively. The maximum of the endothermic peak, most likely due to the thermal degradation of Cu^2+^ and Fe^3+^ complexes of the cr(PVA/Ch)/SQ mats, shifts to lower temperatures (235 °C and 240 °C for the Cu^2+^ and Fe^3+^ complexes of the mats, respectively, Figure 3A(c,d)) compared to that observed in the thermogram of the cr(PVA/Ch)/SQ mats (259 °C, Figure 3A(b)). It can be assumed that the observed changes in the thermal behavior of the cr(PVA/Ch)/SQ mats after immersion in ethanol solution of CuCl_2_ or FeCl_3_ are due to the coordination of Cu^2+^ or Fe^3+^ with the components of the mats.

### 3.4. Water Contact Angle of the Fibrous Materials

The hydrophilic/hydrophobic characteristics of fibrous mats can greatly affect the initial adhesion of cells and their proliferation [56]. Therefore, the water contact angle of the prepared fibrous mats was determined. Digital photographs of the water droplets deposited on the surfaces of the mats are shown in Figure 4. The cr(PVA/Ch) mat was hydrophilic, with a water contact angle of 64.8 ± 3.0°, and the water droplet retained its spherical shape on the surface of the cr(PVA/Ch) mat (Figure 4a). In the case of the cr(PVA/Ch)/SQ mat, the presence of SQ on the fiber surface led to an increase in the hydrophilicity of the mat (the water contact angle value was 38.6 ± 1.8°, Figure 4b). The obtained results showed that the Cu^2+^ and Fe^3+^ complexes of the cr(PVA/Ch)/SQ mats were also hydrophilic. The water drop was immediately absorbed by the mats (their water contact angle was 0°) (Figure 4c,d).

Regarding the potential biomedical application of these fibrous materials in the local treatment of cervical tumors, their hydrophilicity is an important characteristic for achieving a rapid therapeutic effect of a drug. Moreover, the determination of the water contact angle is important for elucidating the relationship between mat composition and the potential application of the mats as wound dressings. It is anticipated that PVA-containing mats, which have low values of water contact angle, will be suitable as wound dressings for the treatment of open wounds, where more exudate leakage is expected, and SQ-containing mats and their Cu^2+^ and Fe^3+^ complexes will be suitable as dressings for infected wounds.

### 3.5. XPS Analysis of the Fibrous Materials

**Figure 5:**
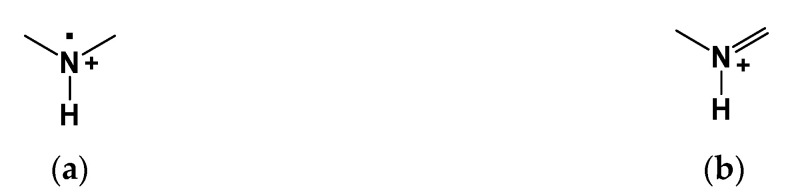
Schematic representation of: polaron species from SQ (**a**) and bipolaron species from SQ (**b**).

The surface composition of the cr(PVA/Ch)/SQ mats or their Cu^2+^ and Fe^3+^ complexes was analyzed by XPS (Supplementary Material, Figures S6–S8). In the detailed C_1s_ spectrum of the cr(PVA/Ch)/SQ mats, the presence of four peaks was observed: at 285.0 eV, assigned to -C-H or -C-C- from PVA, Ch and SQ, and to -C-NH_2_ from Ch; at 286.4 eV for -C-O or -C-OH from PVA and Ch, for -C-N-C=O from Ch, for -C-N, -C-OH and -C-S from SQ and for -C=N from Ch crosslinked with glutaraldehyde; at 288.5 eV for -O-C=O from PVA, for -O-C-O- and -N-C=O from Ch and for -O-C-O- from the acetal groups of crosslinked PVA; and at 290.1 eV, corresponding to π → π * shake-up satellite, characteristic for the SQ ring (Supplementary Material, Figure S6a). In the detailed O_1s_ spectrum (Supplementary Material, Figure S6b), three peaks were identified: at 533.3 eV, assigned to -O-C-O- from Ch and from acetal groups of crosslinked PVA; at 532.5 eV, assigned to -C-O or -C-OH from PVA, to -C-OH from Ch and -C-OH from SQ; and at 532.1 eV, assigned to -O-C=O from PVA. The expanded N_1s_ spectrum (Supplementary Material, Figure S6c) consisted of three components: at 399.4 eV, characteristic for -C-NH_2_ from Ch and for -N=C from crosslinked Ch; at 401.4 eV, assigned to the protonated amino groups (-NH_3_^+^) from Ch and to polaron species from SQ (Figure 5a) [57,58]; and at 402.8 eV, ascribed to bipolaron species from SQ (Figure 5b) [57,58]:

The presence of an S_2p_ peak (Supplementary Material, Figure S6d) consisting of two components S_2p3/2_ at 168.0 eV and S_2p1/2_ at 169.2 eV, characterized by a peak area ratio of 2:1 and a 1.2 eV splitting [57], further confirmed the presence of SQ on the cr(PVA/Ch) fiber surface. 

A confirmation of the successful formation of the Cu^2+^ and Fe^3+^ complexes on the cr(PVA/Ch)/SQ mat surface was obtained from XPS analyses. In comparison to the expanded C_1s_ spectrum of the cr(PVA/Ch)/SQ mats (Supplementary Material, Figure S6a), in the spectra of the Cu^2+^ and Fe^3+^ complexes of the cr(PVA/Ch)/SQ mats, the appearance of a new peak was observed at 287.1 eV, corresponding to -C-O---Cu(Fe) and -C-N---Cu(Fe) (Supplementary Material, Figures S7a and S8a). A decrease in peak intensity at 286.2 eV was also detected. In the detailed O_1s_ spectrum of the Cu^2+^ and Fe^3+^ complexes of the cr(PVA/Ch)/SQ mats, a new peak at low energy (approximately 530.9 eV) appeared, which is most likely due to oxygen atoms coordinated with Cu^2+^ or Fe^3+^ (O---Cu(Fe)) (Supplementary Material, Figures S7b and S8b). No changes in the S_2p_ region were detected, which can be attributed to the fact that the sulfo group of SQ did not coordinate with Cu^2+^ or Fe^3+^ (Supplementary Material, Figures S7d and S8d). The N_1s_ spectrum of these mats, compared to that of the cr(PVA/Ch)/SQ mats, showed the presence of a new component—an intense one at 399.9 eV, corresponding to nitrogen atoms from SQ coordinated with Cu^2+^ or Fe^3+^ (Cu(Fe)---N-C-) (Supplementary Material, Figures S7c and S8c). The appearance of a new peak composed of two components corresponding to Cu_2p1/2_ and Cu_2p3/2_ was detected in the Cu_2p_ region in the XPS spectrum of the Cu^2+^ complex of the cr(PVA/Ch)/SQ mats (Supplementary Material, Figure S7e). This peak was attributed to the coordination of Cu^2+^ with the cr(PVA/Ch)/SQ mats. The observed component Cu_2p3/2_ consisted of a major peak at 933.3 eV and two satellites at 940.9 eV and 944.6 eV. The detected major peak at 933.3 eV was close to the binding energy of 933.1 eV, characteristic for a peak observed by other authors for Cu^2+^ complexes [59]. Figure S8e (Supplementary Material) also shows the high-resolution XPS spectrum of the Fe_2p3/2_ component of the Fe_2p_ region of the Fe^3+^ complex of the cr(PVA/Ch)/SQ mats. The major peak for Fe_2p3/2_ was located at 711 eV. Its binding energy was close to the binding energy of 711.5 eV for the Fe_2p3/2_ peak detected by other authors for Fe^3+^ complexes [60]. The presence of Fe_2p_ peaks confirmed additionally the formation of Fe^3+^ complexes in the surface layer of the cr(PVA/Ch)/SQ mats.

The results obtained from the XPS analyses prove the presence of SQ and of Cu^2+^ or Fe^3+^ complexes on the cr(PVA/Ch) mat’s surface. 

### 3.6. EPR Analysis of Cu^2+^ and Fe^3+^ Complexes of the Fibrous Materials 

**Figure 6:**
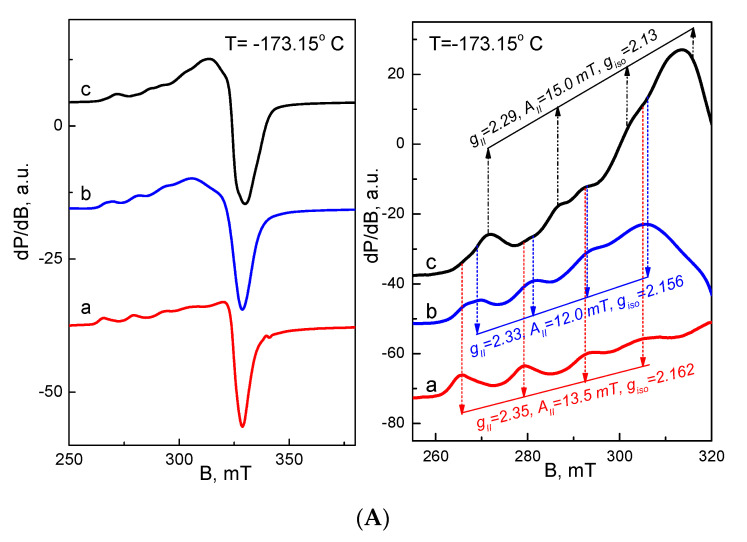

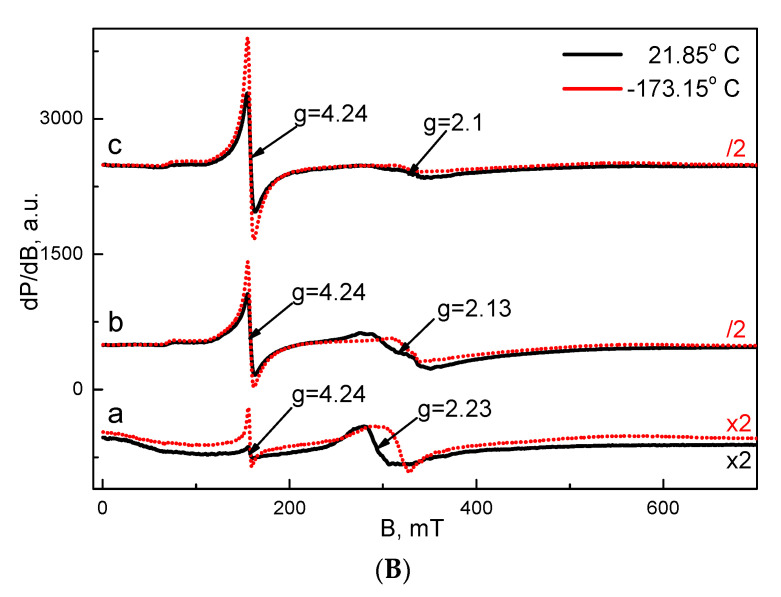
EPR spectra at −173.15 °C of Cu^2+^ (**A**) and at −173.15 °C and 21.85 °C of Fe^3+^ (**B**) complexes of: crPVA mat (a), cr(PVA/Ch) mat (b) and cr(PVA/Ch)/SQ (10 wt% SQ) mat (c).

To obtain insights into the coordination of Cu^2+^ and Fe^3+^ in the complexes of cr(PVA/Ch)/SQ mats, EPR spectroscopy was performed. The EPR analysis was based on the reference complexes, such as the Cu^2+^ and Fe^3+^ complexes of the crPVA and cr(PVA/Ch) mats, respectively (Figure 6). 

The EPR spectrum of the Cu^2+^ complex of the crPVA mat consisted of an anisotropic signal with the following parameters: *g*_||_ = 2.35, *g*_⊥_ = 2.06 and *A*_||_ = 13.5 mT (Figure 6A(a)). These parameters are in good agreement with those previously published [61,62,63] (Table 1). 

The relatively high value of the *g*_||_-component together with the low magnitude of the hyperfine structure (i.e., *A*_||_) revealed the coordination of Cu^2+^ via O atoms in the crPVA mat (Supplementary Material, Scheme S1a). The coordination of Cu^2+^ can be accomplished either between chains of one and the same macromolecule (intramolecular) or between the chains of two macromolecules (intermolecular) of PVA. In the case of the Cu^2+^ complex of the cr(PVA/Ch) mat, an anisotropic signal with *g*- and *A*-values slightly different from those of the Cu^2+^ complex of the crPVA mat was observed: *g*_||_ = 2.33, *g*_⊥_ = 2.06 and *A*_||_ = 12.0 mT (Figure 6A(b)). These parameters implied that the manner of the Cu^2+^ coordination in the complex of the crPVA mat was preserved in the Cu^2+^ complex of the cr(PVA/Ch) mat. Supporting this suggestion, Table 1 lists the EPR parameters previously determined for the complex of Cu^2+^ with Ch. As can be seen in Table 1, there was significant deviation of both *g*_||_ and *A*_||_ for Cu^2+^ ions in the complexes of the cr(PVA/Ch) mat and Ch. Therefore, it can be inferred that the intra- or intermolecular coordination of Cu^2+^ ions with Ch solely was not probable. However, the simultaneous coordination between Cu^2+^ and the chains of both PVA and Ch components of the cr(PVA/Ch) mat might not be excluded [67]. 

The incorporation of the third ligand SQ in the cr(PVA/Ch) mat led to a more significant change in the EPR spectrum of the Cu^2+^ complexes. In the range of the parallel component, several lines with different intensities can be distinguished (Figure 6A(c)). The low-intensity lines were characterized by parameters close to those of the Cu^2+^ complex of the crPVA mat, while the most intensive lines were simulated with the following parameters: *g*_||_ = 2.29, *A*_||_ = 15.0 mT (Figure 6A(a)). It is worth noting that the *g*-components and hyperfine constant were close to those determined by other authors for the isolated Cu^2+^-(8-hydroxyquinoline)_2_.2H_2_O complex (*g*_||_ = 2.287, *g*_⊥_ = 2.066, *A*_||_ = 16.3 mT) [64]. In this complex, the Cu^2+^ ions were coordinated by 2 N- and 2 O-atoms. Based on this comparison, it appears that Cu^2+^ ions are preferentially coordinated by SQ in the cr(PVA/Ch)/SQ mat. Furthermore, it is presumed that Cu^2+^ ions form a complex with two molecules of SQ from the cr(PVA/Ch)/SQ mat through O- and N-atoms, as shown in Scheme S1b (Supplementary Material). 

The EPR spectra of all Fe^3^ complexes displayed two types of signals (Figure 6B). The first signal had a *g*-value of 4.24 and an intensity that increased dramatically in the order of crPVA mat, cr(PVA/Ch) mat and cr(PVA/Ch)/SQ mat (i.e., the relative intensity of 1, 25 to 50, respectively). The signal with *g* = 4.24 was typical for Fe^3+^ ions in rhombic symmetry. This does not allow us to determine univocally the origin of the signal with *g* = 4.24. However, the strong increase in the signal intensity for the Fe^3+^ complexes of the cr(PVA/Ch)/SQ mat hints at the predominant coordination of Fe^3+^ with SQ, most probably through O- and N-atoms [50,68]. In addition, there was a somewhat intensive signal with a g-value of around 2.1 and line width of 65 mT. Contrary to the first signal, the intensity of the second signal was practically the same for the Fe^3+^ complexes of the crPVA, cr(PVA/Ch) and cr(PVA/Ch)/SQ mats. This enables us to attribute, most probably, the second signal with Fe^3+^ coordinated via oxygen atoms of the crPVA component of the mats. 

### 3.7. In Vitro SQ, SQ.Cu^2+^ and SQ.Fe^3+^ Release Studies

**Figure 7:**
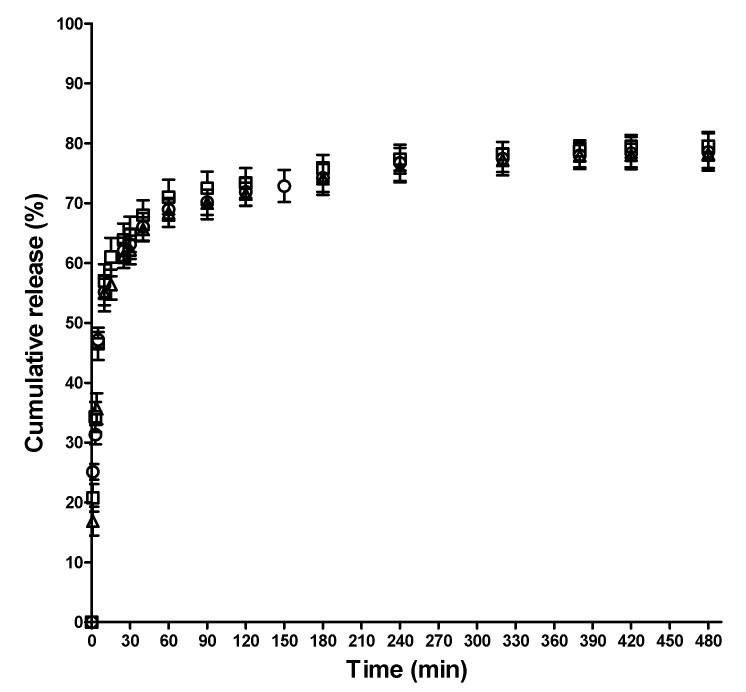
In vitro release profiles of SQ, SQ.Cu^2+^ and SQ.Fe^3+^ from: cr(PVA/Ch)/SQ (10 wt% SQ) mat (□), Cu^2+^ complex of cr(PVA/Ch)/SQ (10 wt% SQ) mat (o) and Fe^3+^ complex of cr(PVA/Ch)/SQ (10 wt% SQ) mat (Δ) in PBS/Tween 40 (99/1 *v*/*v*) at 37 °C, pH 7.4, ionic strength 0.1. Error bars corresponding to the ±SD values calculated based on three replicates for each point.

The in vitro release profiles of SQ, SQ.Cu^2+^ and SQ.Fe^3+^ from the SQ- and SQ.Cu^2+^ (Fe^3+^)-containing mats were examined (Figure 7). 

For all fibrous mats, two stages of release were observed—a first stage of burst release, followed by a second stage of gradual release. The observed initial fast release was most probably due to the diffusion of SQ, SQ.Cu^2+^ or SQ.Fe^3+^, located nearby the surface layer of the fibers. The amount of SQ, SQ.Cu^2+^ and SQ.Fe^3+^ released from the respective mats in the first 25 min was approximately 64%, 62% and 61%, respectively (Figure 7). The total amount of released SQ, SQ.Cu^2+^ and SQ.Fe^3+^ from the SQ- and SQ.Cu^2+^ (Fe^3+^)-containing mats for 480 min was approximately 80%, 79% and 78%, respectively, and remained unchanged for 24 h. The release of SQ, SQ.Cu^2+^ and SQ.Fe^3+^ from the respective mats was mainly influenced by the hydrophilic–hydrophobic characteristics of the fibrous mats. The presence of PVA in the crosslinked fibrous mats, known for its hydrophilicity, is favorable for the release of SQ, SQ.Cu^2+^ and SQ.Fe^3+^ into the buffer medium. The obtained results are in accordance with the data reported in our previous studies for other fibrous systems containing 8-hydroxyquinoline derivatives [36,39,42]. 

A plot of the log of release fraction (M_t_/M_∞_) of SQ, SQ.Cu^2+^ and SQ.Fe^3+^ against the log of time (t) to assess the release mechanism of SQ, SQ.Cu^2+^ and SQ.Fe^3+^ from the respective mats using the Korsmeyer–Peppas model [51] is shown in Supplementary Material, Figure S9. As seen in Figure S9 (Supplementary Material), a linear relationship between log(M_t_/M_∞_) and log(t) for the SQ, SQ.Cu^2+^ and SQ.Fe^3+^ release was observed. The slope of the line was the release exponent (n), which could be used for the determination of the release mechanism. The values of the release exponent (n) for SQ-, SQ.Cu^2+^- and SQ.Fe^3+^-containing mats were 0.41, 0.39 and 0.40, respectively. The R^2^ values for all studied mats were equal to 0.99. The values of n were below 0.45, which indicated that the SQ, SQ.Cu^2+^ and SQ.Fe^3+^ release from the cr(PVA/Ch) mats occurred through the Fickian diffusion mechanism. The obtained results are in conformity with previous reports on the mechanism of drug release from Ch-containing fibrous mats [27,69,70].

In the present study, the prepared SQ-, SQ.Cu^2+^- and SQ.Fe^3+^-containing mats showed an initial burst release of SQ and its complexes, followed by sustained release. The observed release behavior can be favorable for the potential biomedical application of these fibrous materials in the local treatment of cervical tumors or as antibacterial wound dressings. An initial burst of drug release is preferable to achieve an inhibition of tumor cell growth for tumor treatment or for killing the bacteria that are present in wounds. The sustained release is necessary to prevent further proliferation of the surviving tumor or bacterial cells. Thus, the obtained fibrous materials are potential candidates for tumor treatment and for wound dressing applications.

### 3.8. Evaluation of the Antibacterial and Antifungal Activity of the Fibrous Materials

**Figure 8:**
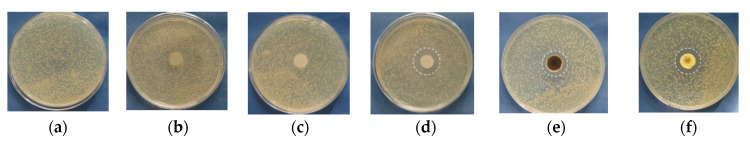

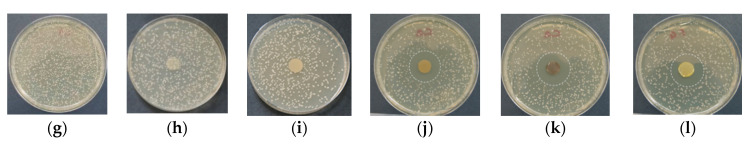
Digital images of the zones of inhibition against *S. aureus* (**a**–**f**) and *C. albicans* (**g**–**l**), detected after 24 h (for *S. aureus*) and 48 h contact (for *C. albicans*) of the fibrous materials with the bacterial or fungal cells. a,g—*S. aureus* or *C. albicans*, (**b**,**h**)—crPVA mat, c,i—cr(PVA/Ch) mat, (**d**,**j**)—cr(PVA/Ch)/SQ (10 wt% SQ) mat, (**e**,**k**)—Fe^3+^ complexes of cr(PVA/Ch)/SQ (10 wt% SQ) mat, (**f**,**l**)—Cu^2+^ complexes of cr(PVA/Ch)/SQ (10 wt% SQ) mat.

The antibacterial and antifungal activities of cr(PVA/Ch)/SQ mats and their Cu^2+^ and Fe^3+^ complexes against Gram-positive bacteria *S. aureus* and fungi *C. albicans* were estimated by performing microbiological tests consisting of determining the diameter of the zone of inhibition around the fibrous materials. It was found that the MIC against bacteria *S. aureus* was the same for SQ and its Cu^2+^ and Fe^3+^ complexes—500 µg/mL—and against fungi *C. albicans*, it was 250 µg/mL, respectively. Digital photographs of the Petri dishes after 24 h or 48 h contact of the fibrous materials with the bacterial and fungal cells are presented in Figure 8. 

The antibacterial and antifungal activity of blank crPVA and cr(PVA/Ch) controls against *S. aureus and C. albicans* was assessed. No inhibition zones were detected around the blank samples (Figure 8b,c,h,i). The tests showed that the SQ-containing mats and their Cu^2+^ and Fe^3+^ complexes had antibacterial and antifungal activity. Moreover, the growth inhibition of fungi *C. albicans* was greater than that of the Gram-positive bacteria *S. aureus* (Figure 8). In these cases, well-defined zones of inhibition of the bacterial and fungal growth were observed. The diameters of the zones of inhibition for the cr(PVA/Ch)/SQ mats and their Fe^3+^ and Cu^2+^ complexes were 25 ± 1.2, 23 ± 1.0 and 24 ± 1.1 mm against *S. aureus* (Figure 8d–f) and 30 ± 2.0, 31 ± 2.4 and 31 ± 1.8 mm against *C. albicans* (Figure 8j–l), respectively. The presence of well-defined zones of inhibition indicated that the incorporated SQ and its Cu^2+^ or Fe^3+^ complexes imparted antibacterial and antifungal activity to the mats. 

### 3.9. Study of the Adhesion of S. aureus Cells to the Surface of the Fibrous Materials

In the present study, the adherence of the bacteria *S. aureus* onto the surface of the fibrous materials was observed by SEM after incubation of the mats in a bacterial suspension for 24 h. These Gram-positive bacteria were used in the test as they are one of the most common types of pathogenic bacteria responsible for secondary wound infections. A large number of bacterial cells were found to adhere to the crPVA mat (Supplementary Material, Figure S10a). In this case, the morphology of the bacterial cells did not change—the cells retained their round shape and had a smooth surface. These observations indicate that the crPVA mat does not inhibit the growth of *S. aureus* and is even a substrate for their adhesion and proliferation and for the further formation of a thick bacterial biofilm. A decrease in the number of adhered bacterial cells was detected upon incorporation of Ch into the crPVA mats (Supplementary Material, Figure S10b). This is most probably due to the antibacterial properties of Ch incorporated into the fibers. On the surface of the cr(PVA/Ch)/SQ mats or their Cu^2+^ and Fe^3+^ complexes, the absence of bacterial cells or the presence of a single bacteria was observed (Supplementary Material, Figure S10c–e). This indicates that pathogenic bacteria have probably been killed by contact with the mats. This tendency towards the prevention of bacterial growth on these electrospun mats can most likely be attributed to the combination of the antibacterial activity of SQ or its complexes incorporated into the fibers and that of Ch. This property is of particular importance because it makes these materials suitable candidates for a number of biomedical applications, e.g., as antibacterial wound dressing materials. 

The results obtained in the performed microbiological tests revealed that the SQ-containing mats and their Cu^2+^ or Fe^3+^ complexes exerted good antibacterial and antifungal properties. The actual mechanism of action of SQ in bacterial and fungal cells has not been completely elucidated yet. It is considered that the antifungal effect of SQ is mainly due to the irreversible damage of the functional integrity of cytoplasmic membranes of the cells, resulting in fungal cell death [44]. It is known that the antibacterial activity of 8-hydroxyquinoline and its derivatives is closely related to their chelating ability towards transition metal ions (Cu^2+^, Fe^2+^, Fe^3+^, etc.) of biological significance. These compounds could bind metallic prosthetic groups of microbial enzymes, thereby leading to the inhibition of enzymatic activity [71,72]. It is also assumed that the charged metal complex of 8-hydroxyquinoline can bind and block the metal-binding sites on bacterial enzymes, which gives rise to the antibacterial effect [72,73]. 

### 3.10. Cytotoxicity of the Fibrous Mats against Human HeLa Tumor Cells

**Figure 9:**
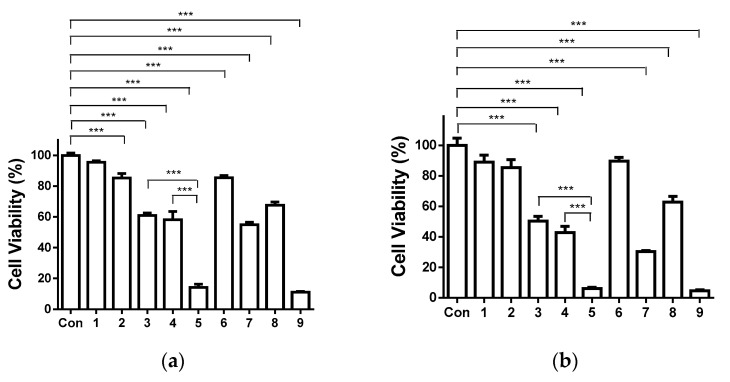

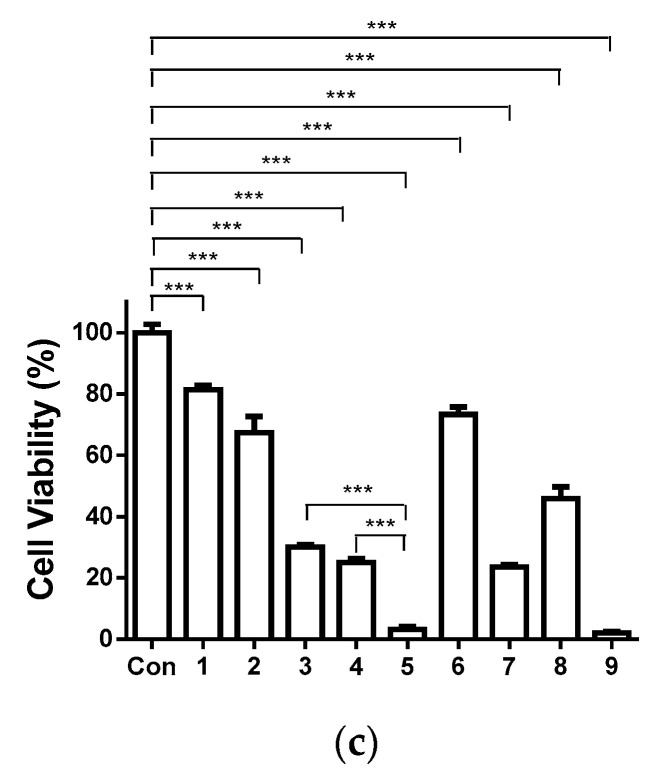
Effect of the different formulations (fibrous mats or solutions) on HeLa tumor cells after 24 (**a**), 48 (**b**) and 72 h (**c**) of incubation. Con—control, HeLa cells; 1—crPVA mat; 2—cr(PVA/Ch) mat; 3—cr(PVA/Ch)/SQ mat; 4—Fe^3+^ complex of cr(PVA/Ch)/SQ mat; 5—Cu^2+^ complex of cr(PVA/Ch)/SQ mat; 6—free Ch; 7—free SQ; 8—SQ.Fe^3+^ and 9—SQ.Cu^2+^. All SQ-containing formulations and their complexes were tested at a concentration of SQ 340 μg/mL of culture medium. *** *p* < 0.001.

SQ and its complexes with metal ions (Cu^2+^, Co^2+^, Ni^2+^, Zn^2+^, Mn^2+^) have been reported to display cytotoxicity towards human tumor cell lines in vitro, such as HeLa and HCT116 [45]. In the present study, the cytotoxic effect of the prepared fibrous mats on human cervical HeLa tumor cells was assessed by MTT assay. After 24 h of incubation (Figure 9a), crPVA mats did not show statistically significant antiproliferative activity—the viability of HeLa cells was 95.6 ± 2.5%. When Ch was incorporated into the crPVA mats, a slight decrease in the viability of HeLa cells was observed (approximately 85.4% of viable cells). For the same time period, the percentage of viable cells decreased significantly to 61.0 ± 3.8% and 58.1 ± 10.9% for the SQ-containing cr(PVA/Ch) mats and their Fe^3+^ complexes, respectively. The Cu^2+^ complexes of the cr(PVA/Ch)/SQ mats showed the highest antiproliferative activity against HeLa cells—cell viability considerably decreased to 14.2 ± 4.6%, respectively. At the 48th hour of incubation (Figure 9b), the decrease in the proliferative activity of HeLa cells in SQ-containing mats and their complexes was higher than that observed at the 24th hour of incubation. The percentage of cell viability was statistically significantly reduced to 50.4 ± 7.4, 42.9 ± 9.1, 6.2 ± 1.8 for cr(PVA/Ch)/SQ mats and their Fe^3+^ and Cu^2+^ complexes, respectively. At the 72nd hour of incubation (Figure 9c), the strongest antiproliferative effect was observed in SQ-containing mats and their complexes. It should be noted that the Cu^2+^ complexes of the cr(PVA/Ch)/SQ mats (3.2 ± 2.2% viable cells) displayed higher cytotoxicity than the cr(PVA/Ch)/SQ mats (30.0 ± 2.1% viable cells) and their Fe^3+^ complexes (25.1 ± 2.3% viable cells). Moreover, the percentage of viable HeLa cells for the Cu^2+^ complexes of SQ-containing mats was close to that for the SQ.Cu^2+^ solution (2.1 ± 0.9%). 

**Figure 10:**
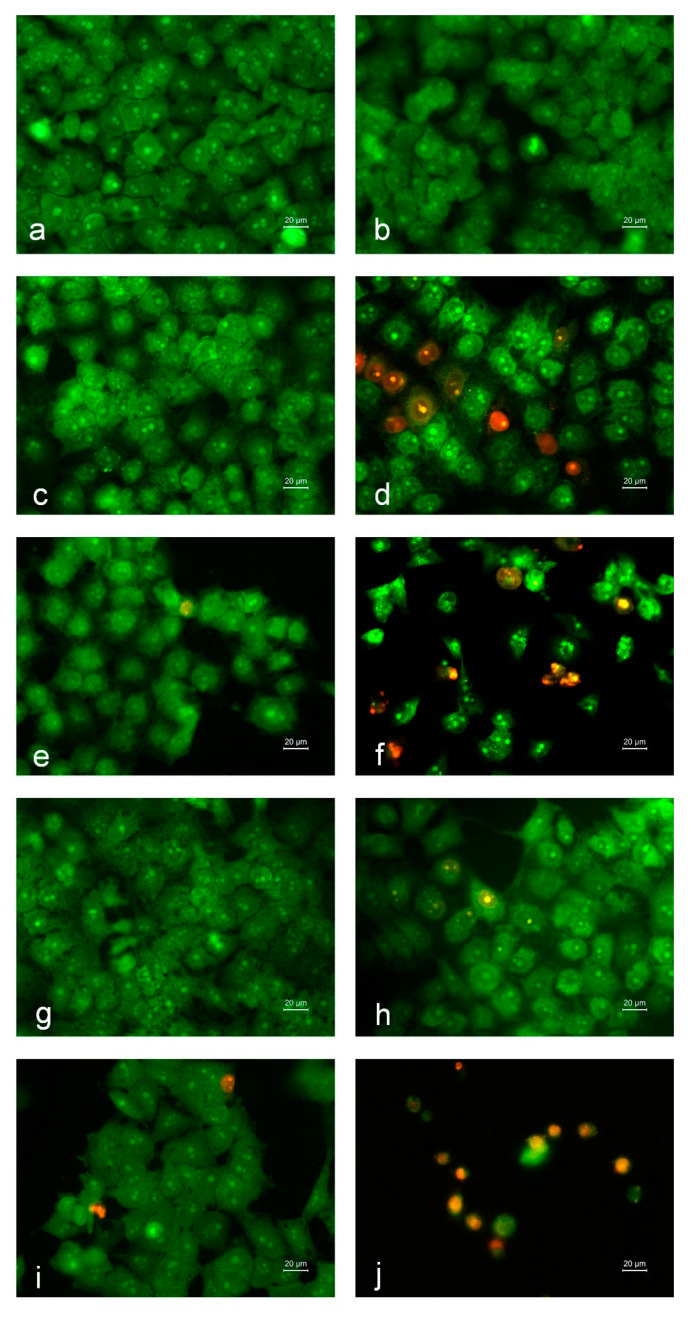
Fluorescence micrographs of AO and EtBr double-stained HeLa tumor cells incubated in the presence of different formulations (fibrous mats or solutions) for 24 h. Cells after incubation with: (**a**) untreated HeLa cells, (**b**) crPVA mat, (**c**) cr(PVA/Ch) mat, (**d**) cr(PVA/Ch)/SQ mat, (**e**) Fe^3+^ complex of cr(PVA/Ch)/SQ mat, (**f**) Cu^2+^ complex of cr(PVA/Ch)/SQ mat, (**g**) aqueous solution of Ch, (**h**) aqueous solution of SQ, (**i**) solution of SQ.Fe^3+^ and (**j**) solution of SQ.Cu^2+^; scale bar = 20 μm. All SQ-containing formulations and their complexes were tested at a concentration of SQ 340 μg/mL of culture medium.

Experiments were performed to elucidate the extent to which inhibition of HeLa tumor cell proliferation is achieved via apoptosis. Staining with a mixture of fluorescent dyes (AO and EtBr) (1:1 *w*/*w*) (Figure 10) was performed on HeLa cells cultured for 24 h in the presence of the studied fibrous mats. After staining, the cells were analyzed with a fluorescence microscope. Figure 10 shows HeLa cells with morphological changes that occurred as a result of the interaction with the studied mats. In the case of staining of untreated HeLa cells (negative control), cells with pale green nuclei and bright yellow-green nucleoli were dominant (Figure 10a). These cells showed normal morphology. Accumulations of orange granules located perinuclearly were also observed. The morphology of HeLa cells cultured on contact with crPVA mats was also normal (Figure 10b). When incubating the cells in the presence of cr(PVA/Ch) mats, round cells with bright green nuclei with condensed chromatin and blebbing of the cell membrane (morphological signs of early apoptosis) were detected (Figure 10c). Similar early apoptotic changes in morphology were observed in HeLa cells that were in contact with Ch solutions (Figure 10g). When cells were treated with cr(PVA/Ch)/SQ mats or with their Fe^3+^ and Cu^2+^ complexes, or when the cells were placed in contact with free SQ or its complexes, the presence of different degrees of morphological changes in the cells was observed, characteristic of early or late apoptosis (Figure 10d–f,h–j). A cytotoxic effect occurred when using cr(PVA/Ch)/SQ mats or their Fe^3+^ complexes, a solution of SQ or its Fe^3+^ complex (Figure 10d,e,h,i). In these cases, the monolayer of cells was disrupted; along with cells with signs of early apoptosis (bright green nuclei with condensed chromatin in the form of dense green areas), cells with signs of late apoptosis were observed—round cells with bright orange nuclei with condensed and aggregated chromatin, with nucleus fragmentation and the appearance of apoptotic bodies. These changes were significantly greater in HeLa cells treated with the Cu^2+^ complex of the cr(PVA/Ch)/SQ mats or with a solution of SQ.Cu^2+^ (Figure 10f,j). In this case, single cells were detected, the predominant part of which had signs of late apoptosis—round cells of different sizes, with wrinkled nuclei and cytoplasm, loss of orange granules in the cytoplasm and the presence of dead, destructured cells with pyknotic nuclei. 

The obtained results revealed that the Cu^2+^ complexes of the cr(PVA/Ch)/SQ mats showed strong cytotoxicity towards HeLa cells. The SQ-containing mats and their Fe^3+^ complexes displayed a weaker cytotoxic effect.

In addition, DAPI staining was applied to examine changes in the nuclei of HeLa cells in vitro. Untreated HeLa cells had intact nuclei and were slightly oval in shape, uniform in size, with smooth edges and evenly distributed chromatin (Supplementary Material, Figure S11a). In this case, cell nuclei were also observed in different stages of mitosis. HeLa tumor cells that were in contact with crPVA mats showed nuclei with similar morphology to that of control tumor cells (Supplementary Material, Figure S11b). Close to that of the control was the morphological characteristics of the nuclei of HeLa cells cultured in the presence of cr(PVA/Ch) mat (Supplementary Material, Figure S11c) or treated with Ch solution (Supplementary Material, Figure S11g). In this case, a coarser and inhomogeneously colored chromatin was observed in the nucleus. After treatment of HeLa cells with cr(PVA/Ch)/SQ mats, with their complexes and with free SQ and its complexes, morphological changes occurred in the nuclei of cells typical of apoptosis, such as nuclear polymorphism (nuclei different in shape and size), condensation of chromatin, nuclei pyknosis, nuclei fragmentation and the formation of apoptotic bodies (Supplementary Material, Figure S11d–f,h–j). These changes were more evident in HeLa cells that were in contact with the Cu^2+^ complex of the cr(PVA/Ch)/SQ mats or with a solution of SQ.Cu^2+^ (Supplementary Material, Figure S11f,j) and less obvious for cr(PVA/Ch)/SQ mats and their Fe^3+^ complexes or for SQ and SQ.Fe^3+^ solutions (Supplementary Material, Figure S11d,e,h,i). The results obtained are in agreement with the data obtained from the MTT test. These results showed that SQ-containing mats and their complexes with Fe^3+^ and Cu^2+^ exhibited good cytotoxicity against the HeLa cells. The induced changes in the cell and nuclear morphology of HeLa cells indicate that cr(PVA/Ch)/SQ fibrous mats and their Fe^3+^ and Cu^2+^ complexes cause death of HeLa cells, and this occurs via the induction of apoptosis.

## 4. Conclusions

In the present study, for the first time, SQ-containing fibrous materials were prepared by electrospinning. Cu^2+^ and Fe^3+^ complexes of the fibrous mats were obtained by treating crosslinked PVA/Ch/SQ mats with CuCl_2_ or FeCl_3_ solution. The performed DSC analyses have demonstrated that SQ, SQ.Cu^2+^ and SQ.Fe^3+^ incorporated into the fibrous materials were in the amorphous state, which is favorable for utilization in drug dosage forms. The EPR analyses indicated that in the complexes of the cr(PVA/Ch)/SQ mat, the Cu^2+^ or Fe^3+^ ions are preferentially coordinated by SQ, most likely forming mononuclear complexes. In addition, there are some Cu^2+^ and Fe^3+^ ions coordinated by oxygen atoms only, as in the case of the Cu^2+^ (Fe^3+^) complex of PVA. The presence of PVA in the crosslinked fibrous mats promoted the release of SQ and its complexes. The SQ-containing mats and their Cu^2+^ and Fe^3+^ complexes were effective in inhibiting the growth of the Gram-positive bacteria *S. aureus* and fungi *C. albicans*. These materials had the capability of suppressing the adhesion of pathogenic *S. aureus* bacteria. In addition, cr(PVA/Ch)/SQ mats and their complexes exerted good antitumor effects against HeLa tumor cells due to the induction of apoptosis. The prepared novel fibrous materials are promising for wound dressing applications and for application in the local treatment of cervical tumors.

## Figures and Tables

**Table 1:** EPR data for Cu^2+^ complexes with different ligands—SQ, PVA and Ch.

Cu^2+^ Complex	*g*_II_	*A*_II_ (mT)	*g*_⊥_
Cu^2+^-SQ (this work)	2.290	15.0	2.050
* Cu^2+^-8-hydroxyquinoline [64]	2.287	16.3	2.066
Cu^2+^-PVA (this work)	2.350	13.5	2.069
* Cu^2+^-PVA [62]	2.322	10.8	2.069
* Cu^2+^-PVA [63]	2.320	11.2	2.060
Cu^2+^-PVA, Ch (4O)(this work)	2.330	12.0	2.069
* Cu^2+^-Ch, (4N) [65]	2.244	17.5	2.069
* Cu^2+^-Ch, (2N,2O) [66]	2.254	16.2	2.061

* Literature data.

## Data Availability

The data presented in this study are available on request from the corresponding author.

## Supplementary Materials

The following are available online at https://www.mdpi.com/article/10.3390/polym13162690/s1, Table S1: Dynamic viscosity and conductivity of the spinning solutions, Figure S1: SEM micrographs of electrospun mats of PVA and PVA/Ch, Figure S2: Equilibrium swelling degree of crosslinked SQ-containing mat in acetate buffer of pH 4.5 versus time, Figure S3: ATR-FTIR spectra of PVA/Ch and cr(PVA/Ch) mats, Figure S4: ATR-FTIR spectra of SQ, SQ-containing mats and their complexes, Figure S5: DSC thermogram of crPVA mat, Figure S6: XPS peak fittings for crosslinked SQ-containing mat, Figure S7: XPS peak fittings for Cu^2+^ complex of crosslinked SQ-containing mat, Figure S8: XPS peak fittings for Fe^3+^ complex of crosslinked SQ-containing mat, Scheme S1: Schematic representation of coordination of Cu^2+^ in crosslinked SQ-containing mat, Figure S9: Korsmeyer–Peppas model of SQ, SQ.Cu^2+^(Fe^3+^) release from SQ-containing mats and their complexes, Figure S10: SEM micrographs of mats that have been incubated in *S. aureus* cell culture, Figure S11: Fluorescence microscopic images of HeLa tumor cells stained with DAPI after treatment with crPVA mat, cr(PVA/Ch) mat, Ch, crosslinked SQ-containing mats and their complexes or with SQ and its complexes.
