# Supplementary material for: 8-Hydroxyquinoline-5-Sulfonic Acid-Containing Poly (Vinyl Alcohol)/Chitosan Electrospun Materials and Their Cu2+ and Fe3+ Complexes: Preparation, Antibacterial, Antifungal and Antitumor Activities
Source: Polymers (Basel). 2021 Aug 12;13(16):2690. doi: 10.3390/polym13162690 (PMC8400372; doi:10.3390/polym13162690)
Supplement: Supplementary file 1 [file polymers-13-02690-s001.zip › polymers-1324119-supplementary.pdf]

Electronic Supplementary Material

# 8-Hydroxyquinoline-5-sulfonic acid-containing poly(vinyl alcohol)/chitosan electrospun materials and their $Cu^{2+}$ and $Fe^{3+}$ complexes: Preparation, antibacterial, antifungal and antitumor activities

Milena Ignatova[1,*], Nevena Manolova[1,*], Iliya Rashkov[1], Nadya Markova[2], Rositsa Kukeva[3], Radostina Stoyanova[3], Ani Georgieva[4] and Reneta Toshkova[4]

[1]*Laboratory of Bioactive Polymers, Institute of Polymers, Bulgarian Academy of Sciences, Acad. G. Bonchev St, Bl. 103A, BG-1113 Sofia, Bulgaria*

[2]*Institute of Microbiology, Bulgarian Academy of Sciences, Acad. G. Bonchev Bl. 26, BG-1113 Sofia, Bulgaria*

[3]*Institute of General and Inorganic Chemistry, Bulgarian Academy of Sciences, Acad. G. Bonchev St, bl. 11, BG-1113 Sofia, Bulgaria*

[4]*Institute of Experimental Morphology, Pathology and Anthropology with Museum, Bulgarian Academy of Sciences, Acad. G. Bonchev St, bl. 25, BG-1113 Sofia, Bulgaria*

## Preparation of the complexes SQ.$Cu^{2+}$and SQ.$Fe^{3+}$

The complex with a molar ratio of SQ and $Cu^{2+}$ 2:1 (further denoted as SQ.$Cu^{2+}$) was prepared by procedure described in details elsewhere [1]. Briefly, a solution of NaOH (234 mg, 4 mmol) in ethanol was added to a solution of SQ (450 mg, 2 mmol) in 120 mL of ethanol. The mixture was stirred for 2h at room temperature. After that, the pH of the solution was adjusted to 7 by dropwise addition of 2M NaOH solution. A solution of $CuCl_2$ (170 mg, 1 mmol) in ethanol (5 mL) was added and the reaction mixture was stirred for 20 min at room temperature. The green precipitate that formed was filtered, washed with ethanol followed by ether and then vacuum dried at 80 °C. Yield of SQ.$Cu^{2+}$ – 70%. ATR-FTIR, ν/$cm^{-1}$ :3445-3356 (ν(O-H)), 3088-3061 (ν(C-H)), 1582, 1501, 1466 (ν(C=C)), 1599 (ν(C=N)), 1375 (δ(C-H)), 1242 (ν(C-O)), 1179 (δ(SOH)), 1040 (ν(O=S=O)).

The complex with a molar ratio of SQ and $Fe^{3+}$ 3:1 (further denoted as SQ.$Fe^{3+}$) was synthesized according to a known procedure [2]. Briefly, to a solution of SQ (675 mg, 3.0 mmol) in 30 mL 0.1 M NaOH, a solution of $FeCl_3$ (162 mg, 1.0 mmol) in 10 mL water was added dropwise. The pH of the solution was adjusted to 4~5 by adding 1 mol/L HCl. The mixture was refluxed for 2h, then the water was distilled off and the residue was washed with water-ethanol mixture (1:9), recrystallized from anhydrous ethanol and vacuum dried at 80

$^{o}$C. Yield of SQ.$Fe^{3+}$ – 80%. ATR-FTIR, ν/$cm^{-1}$: 3100-3044 (ν(C-H)), 1624, 1607, 1553, 1500, 1462 (ν(C=C)), 1593 (ν(C=N)), 1383 (δ(C-H)), 1254 (ν(C-O)), 1219 (δ(SOH)), 1034 (ν(O=S=O)).

**Determination of the minimum inhibitory concentration of SQ and its $Cu^{2+}$ and $Fe^{3+}$complexes**

The minimum inhibitory concentration (MIC) of SQ and its $Cu^{2+}$ and $Fe^{3+}$complexes was determined for Gram-positive bacteria *S.aureus* 3359 and for fungi *C. albicans* 74, respectively. *S.aureus* was cultivated overnight in Tryptic Soy Agar (TSA, Becton Dickinson) at 37◦C, while *C.albicans* - in Sabouraud Dextrose Agar (SDA, Becton Dickinson) at 37$^{o}$C for 48h. The inoculum suspensions of both *S.aureus* and *C.albicans* cultures were prepared with fresh broth media as followed: for *S.aureus* with Tryptic Soy Broth (TSB, Becton Dickinson) and for *C.albicans* with Sabouraud Dextrose Broth (SDB, Becton Dickinson) to concentration of 1 X $10^{5}$ cells/mL (determined optically using a standard). The tested SQ was dissolved in distilled water at an initial concentration of 0.004 g/mL. The SQ.$Cu^{2+}$ and SQ.$Fe^{3+}$ were dissolved in DMSO at an initial concentration of SQ of 0.004 g/mL After two-fold falling serial dilutions of tested SQ, SQ.$Cu^{2+}$ and SQ.$Fe^{3+}$ in the respective broths (TSB for *S.aureus* and SDB for *C.albicans*), the equal aliquots from bacterial and fungal suspensions were added to the every tube of the dilution series. Readings were made after 24h incubation of *S.aureus* and 48 h of *C.albicans* at 37°C by visually comparing the turbidity of each tube with that of the control tube (without SQ or its $Cu^{2+}$ and $Fe^{3+}$complexes).

**ATR-FTIR characteristic bands of the non-crosslinked PVA/Ch mats**

The ATR-FTIR spectrum of non-crosslinked PVA/Ch mats (Supplementary Material**,** Fig. S3a) showed absorption characteristic bands at 3302 $cm^{-1}$ (O-H and N-H stretching vibrations of PVA and Ch), at 2938 and 2909 $cm^{-1}$ (aliphatic C-H stretching vibrations), at 1734 $cm^{-1}$ and 1717 $cm^{-1}$ (C=O stretching vibrations from residual vinyl acetate repeating units in PVA and from residual acetyl groups in Ch), at 1090 $cm^{-1}$ (C-O-C stretching vibrations of PVA). Bands were also recorded at 1653 $cm^{-1}$ assigned to amide I of the polysaccharide structure of Ch and C-O stretching vibrations of acetyl groups of Ch, as well as at 1593 $cm^{-1}$ due to amide II of Ch (Supplementary Material, Fig. S3a). The appearance of a band at 1558 $cm^{-1}$ ascribed to the N-H bending vibration of the $–NH_3^{+}$ group of Ch was recorded.

**ATR-FTIR characteristic bands of SQ**

In the ATR-FTIR spectrum of the free SQ (Supplementary Material**,** Fig. S4a) bands for stretching vibrations of the phenolic O-H group (3443 $cm^{-1}$ and 3383 $cm^{-1}$), bands for C-H stretching vibrations of the SQ ring (3086 $cm^{-1}$ and 3065 $cm^{-1}$), bands for stretching vibration of the SQ ring (1624 $cm^{-1}$, 1605 $cm^{-1}$, 1553 $cm^{-1}$ and 1497 $cm^{-1}$), a C=N stretching vibration band (1589 $cm^{-1}$), a band for C-H bending vibration (1381 $cm^{-1}$), a band for C-O stretching vibration (1261 $cm^{-1}$), bands for in-plane and out-of-plane CH deformations of the poly-substituted phenyl ring (945 $cm^{-1}$ и 858 $cm^{-1}$) and a band for out-of-plane CH deformation modes (770 $cm^{-1}$) were detected [3]. Two bands were also registered at 1038 $cm^{-1}$ and 1177 $cm^{-1}$ characteristic of $SO_2$ stretching vibrations and SOH bending vibrations from the sulfo group of SQ, respectively.

**ATR-FTIR characteristic bands of cr(PVA/Ch)/SQ mat**

In the ATR-FTIR spectrum of cr(PVA/Ch)/SQ mat (Fig. S4c), a 6 $cm^{-1}$ shift of the band for C-O-C stretching vibrations of PVA toward the higher wavenumber to 1096 $cm^{-1}$ was detected compared to the spectrum of the non-crosslinked PVA/Ch/SQ mat (1090 $cm^{-1}$) (Fig. S4b). This is most likely due to the formation of C-O-C bonds from acetal and ether groups, as a result of the interaction of -OH groups of PVA with aldehyde groups of the crosslinking agent. A decrease in the ratio of the band intensities at 3327 $cm^{-1}$ and 2941 $cm^{-1}$ in the spectrum of the cr(PVA/Ch)/SQ mat was also recorded compared to that in the spectrum of the non-crosslinked PVA/Ch/SQ mat, which might be attributed to the consumption of hydroxyl and amino groups during crosslinking of PVA and Ch.

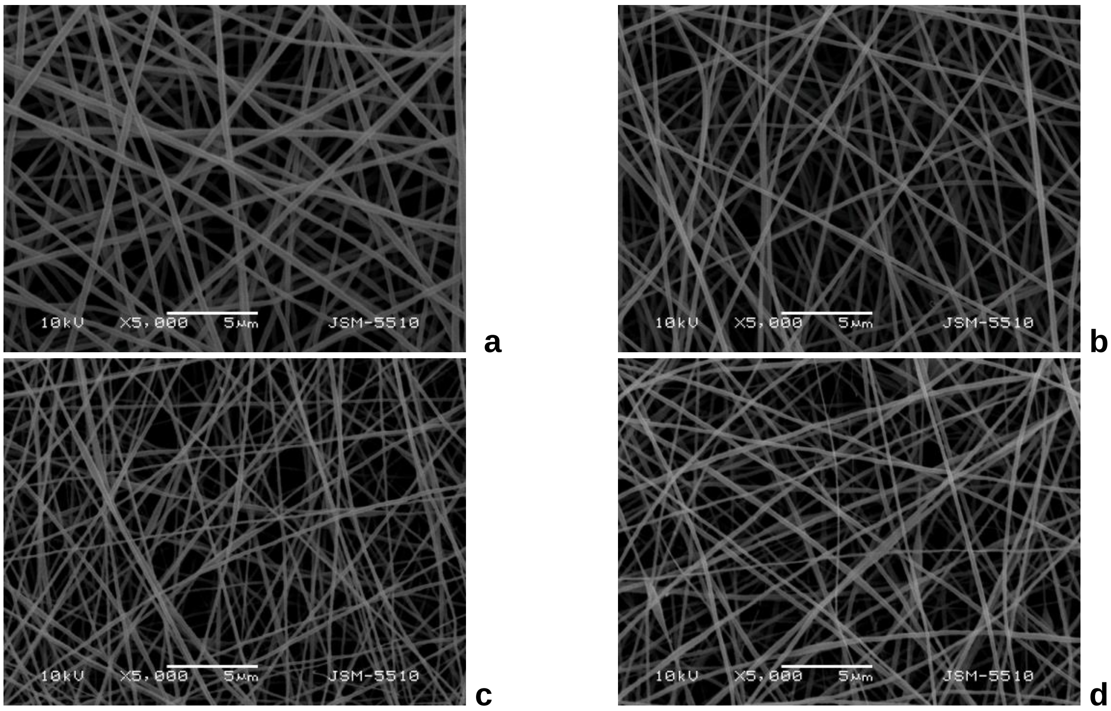


**Figure S1**. SEM micrographs of electrospun mats of: PVA (a), PVA/Ch (9:1 w/w) (b), PVA/Ch (8:2 w/w) (c), PVA/Ch (7:3 w/w) (d); magnification × 5000.

**Table S1**. Dynamic viscosity (η) and conductivity (σ) of the spinning solutions.

| Electrospun mats | $\eta$ (cP) | σ (μS/cm) |
|---|---|---|
| PVA | 400 | 484 |
| PVA/Ch (9/1 w/w) | 800 | 2370 |
| PVA/Ch (8/2 w/w) | 1530 | 2500 |
| PVA/Ch (7/3 w/w) | 2000 | 3000 |
| PVA/Ch/SQ (5 wt% SQ) | 1530 | 2140 |
| PVA/Ch/SQ (10 wt% SQ) | 1525 | 1840 |

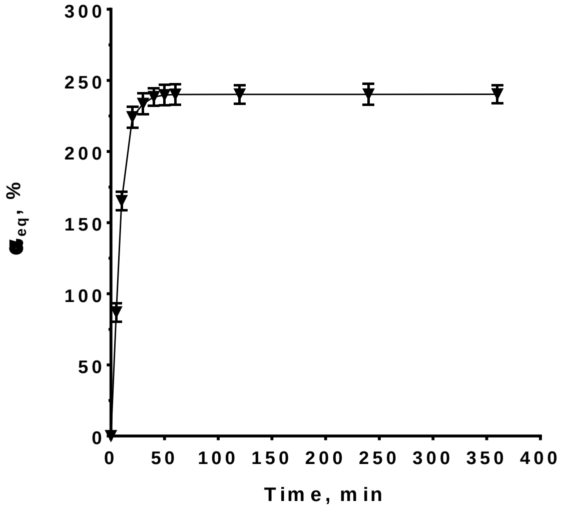


**Figure S2**. Equilibrium swelling degree ($\alpha_{eq}$), of cr(PVA/Ch)/SQ (10wt% SQ) mat (▼) in acetate buffer of pH 4.5 versus time.

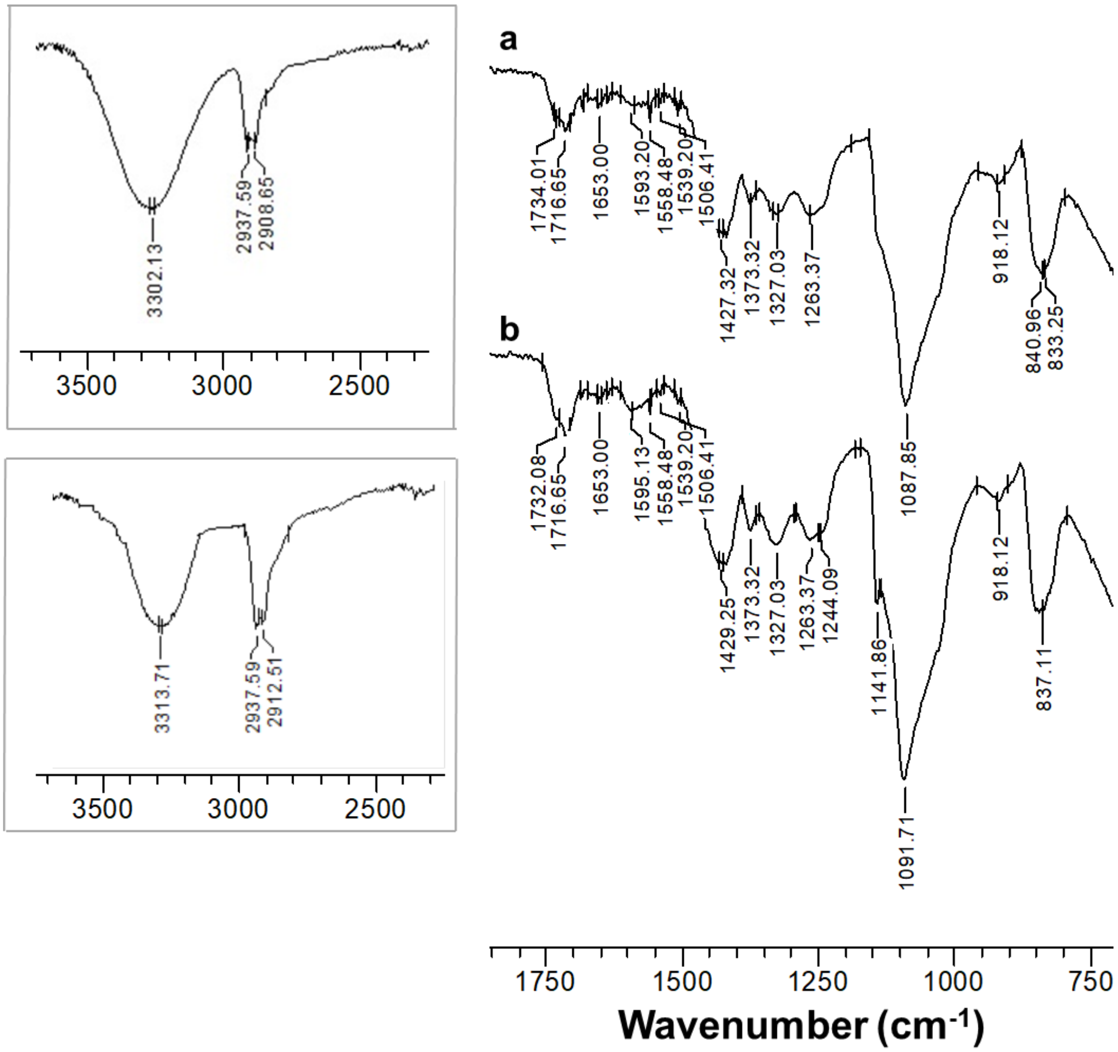


**Figure S3**. ATR-FTIR spectra of: (a) PVA/Ch mat and (b) cr(PVA/Ch) mat in the range from 1750 to 750 cm$^{-1}$ and from 3500 to 2500 cm$^{-1}$ (inset).

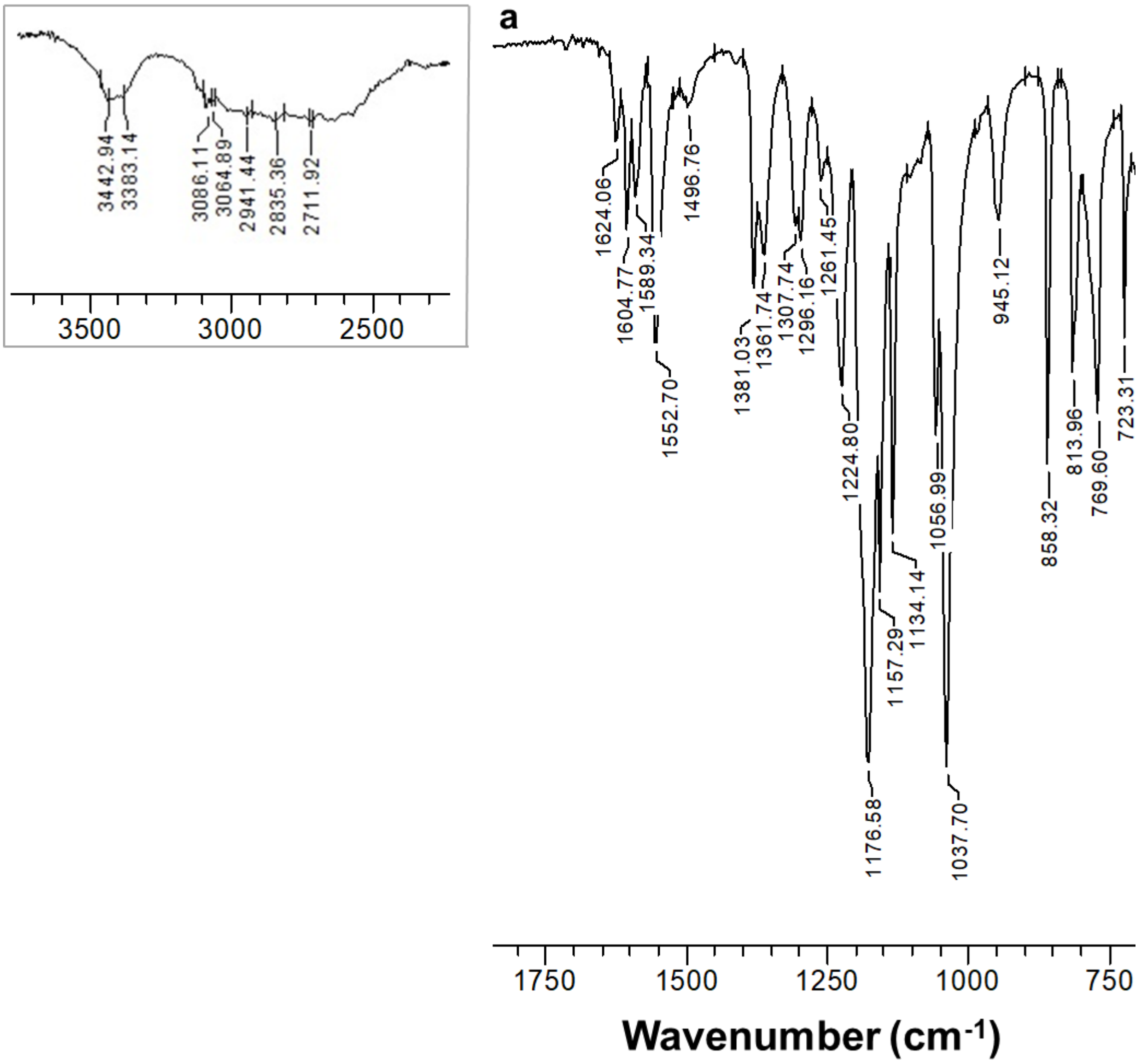
a
3442.94
3383.14
3086.11
3064.89
2941.44
2835.36
2711.92
3500
3000
2500
1624.06
1604.77
1589.34
1552.70
1496.76
1381.03
1361.74
1307.74
1296.16
1261.45
1224.80
1176.58
1157.29
1134.14
1056.99
1037.70
945.12
858.32
813.96
769.60
723.31
1750
1500
1250
1000
750
Wavenumber (cm-1)

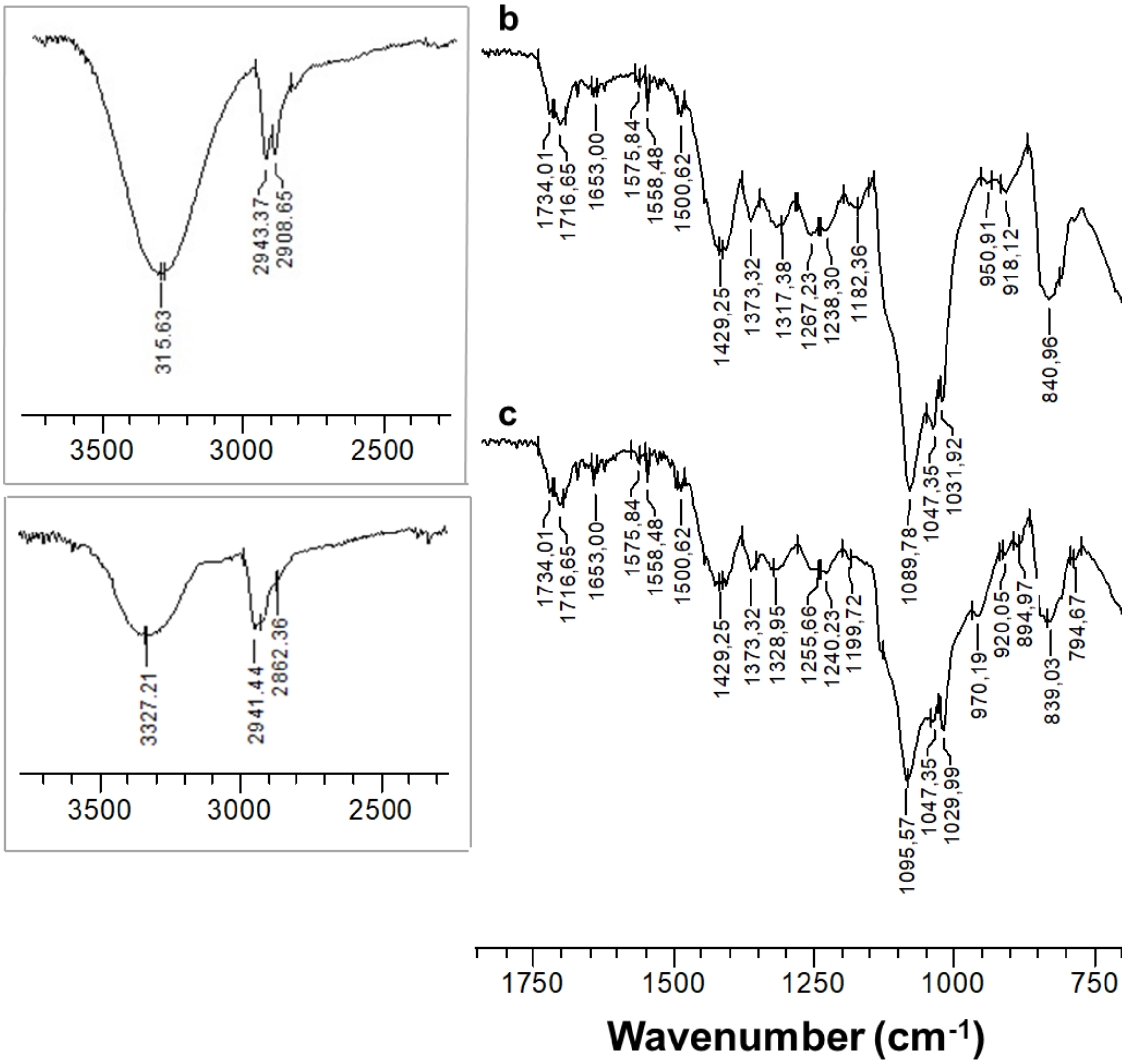
b
c
315.63
2943.37
2908.65
3327.21
2941.44
2862.36
3500
3000
2500
1734,01
1716,65
1653,00
1575,84
1558,48
1500,62
1429,25
1373,32
1317,38
1267,23
1238,30
1182,36
1089,78
1047,35
1031,92
950,91
918,12
840,96
1328,95
1255,66
1240,23
1199,72
1095,57
1029,99
970,19
920,05
894,97
839,03
794,67
1750
1500
1250
1000
750
Wavenumber (cm-1)

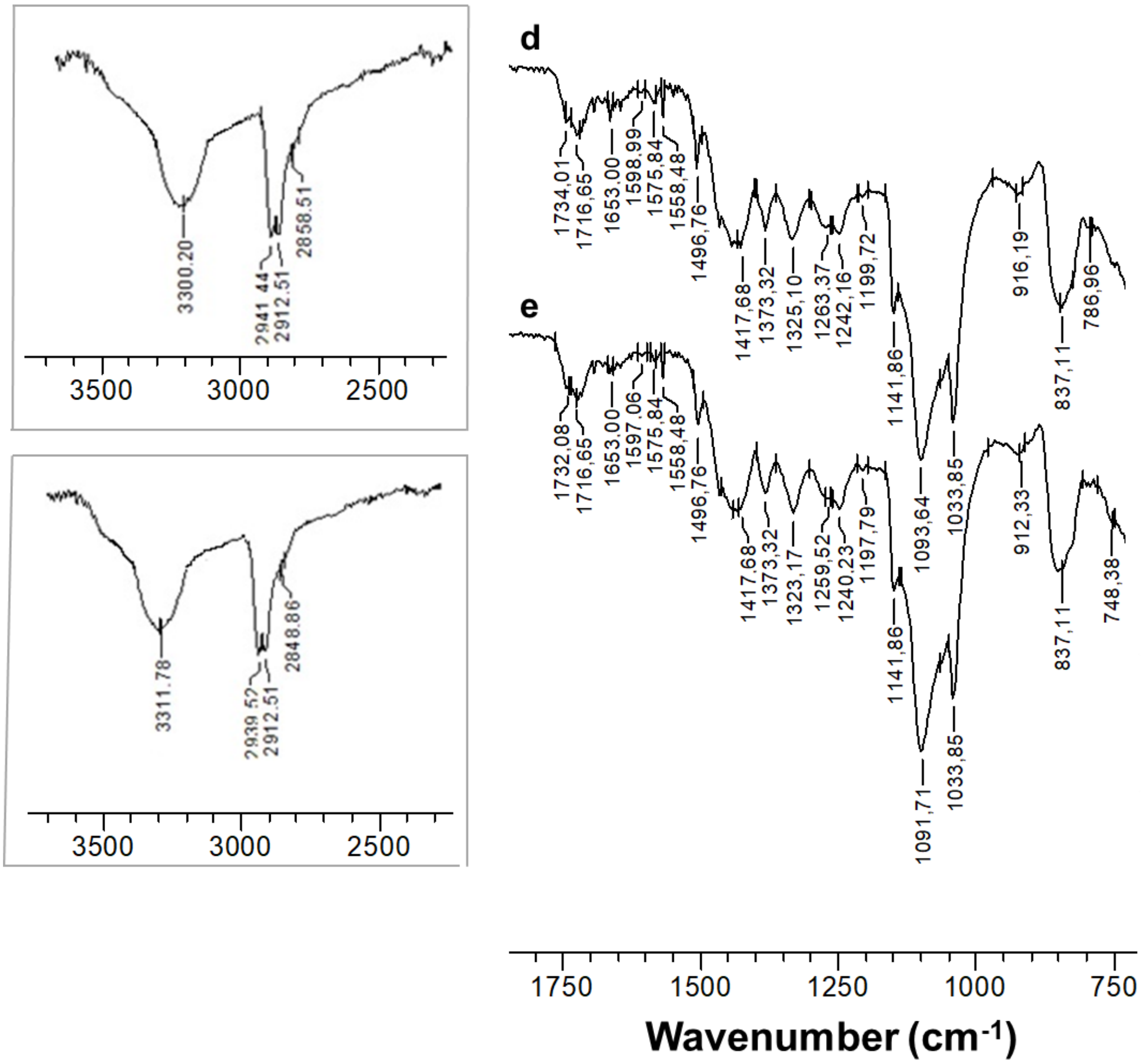


**Figure S4**. ATR-FTIR spectra of: (a) SQ, (b) PVA/Ch/SQ (10wt% SQ) mat, (c) cr(PVA/Ch)/SQ (10wt% SQ) mat, (d) $Cu^{2+}$complex of cr(PVA/Ch)/SQ (10wt% SQ) mat and (e) $Fe^{3+}$complex of cr(PVA/Ch)/SQ (10wt% SQ) mat in the range from 1750 to 750 $cm^{-1}$ and from 3500 to 2500 $cm^{-1}$ (inset).

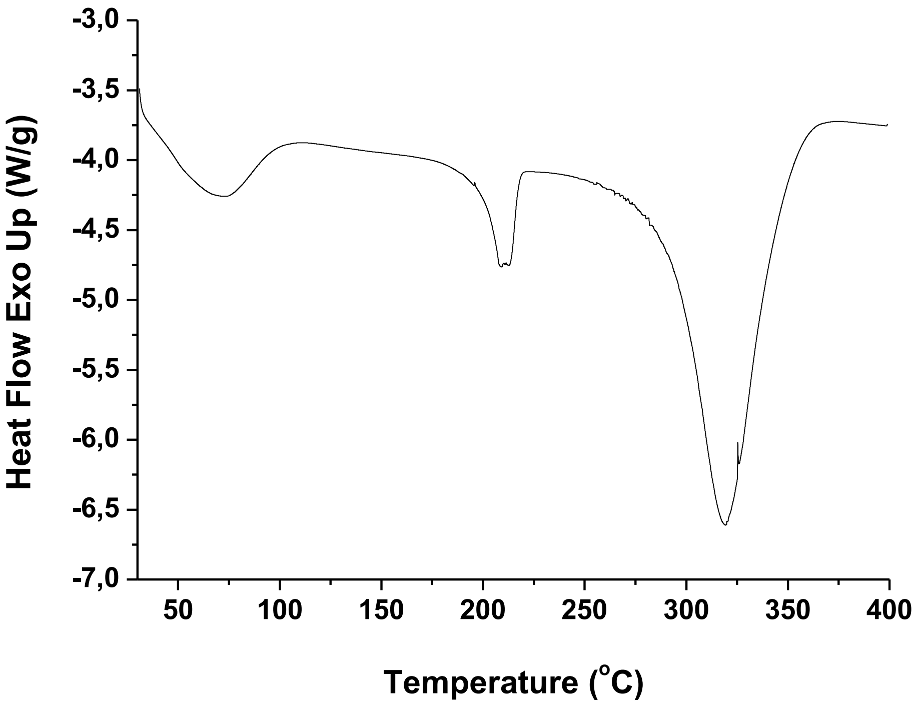


**Figure S5**. DSC thermogram of crPVA mat.

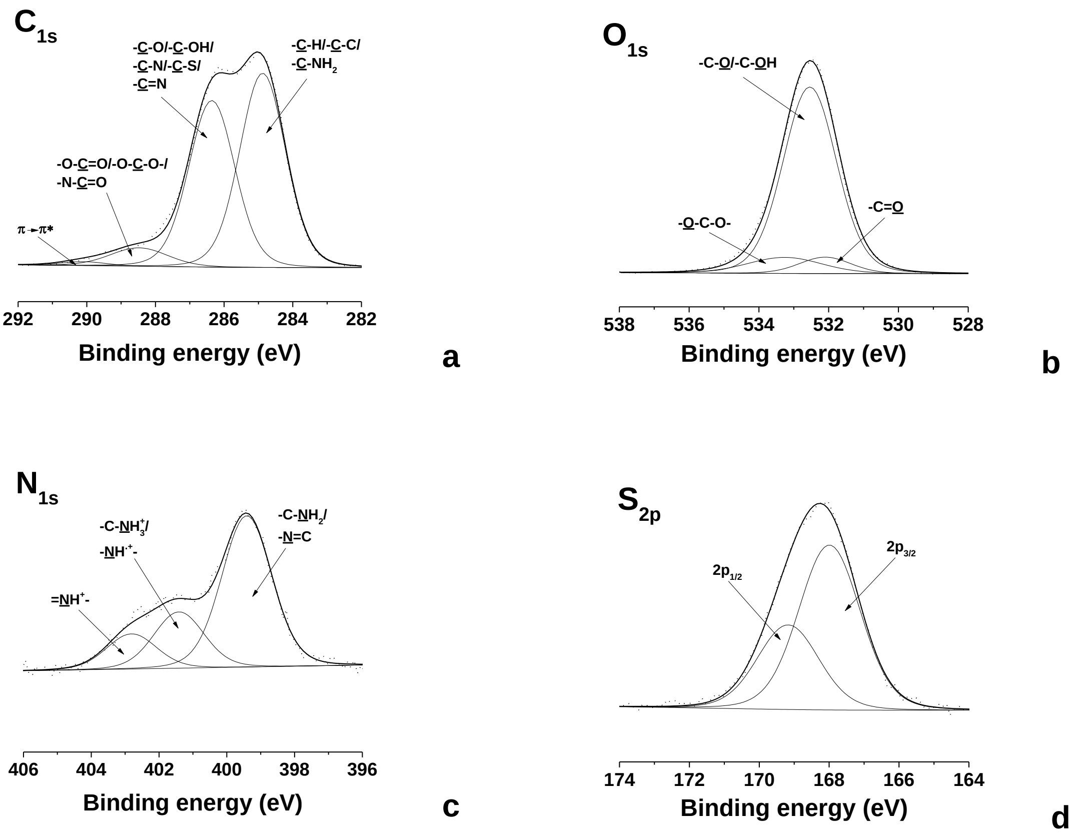


**Figure S6**. XPS peak fittings for cr(PVA/Ch)/SQ (10wt% SQ) mat [$C_{1s}$ (a), $O_{1s}$ (b), $N_{1s}$ (c), $S_{2p}$ (d)].

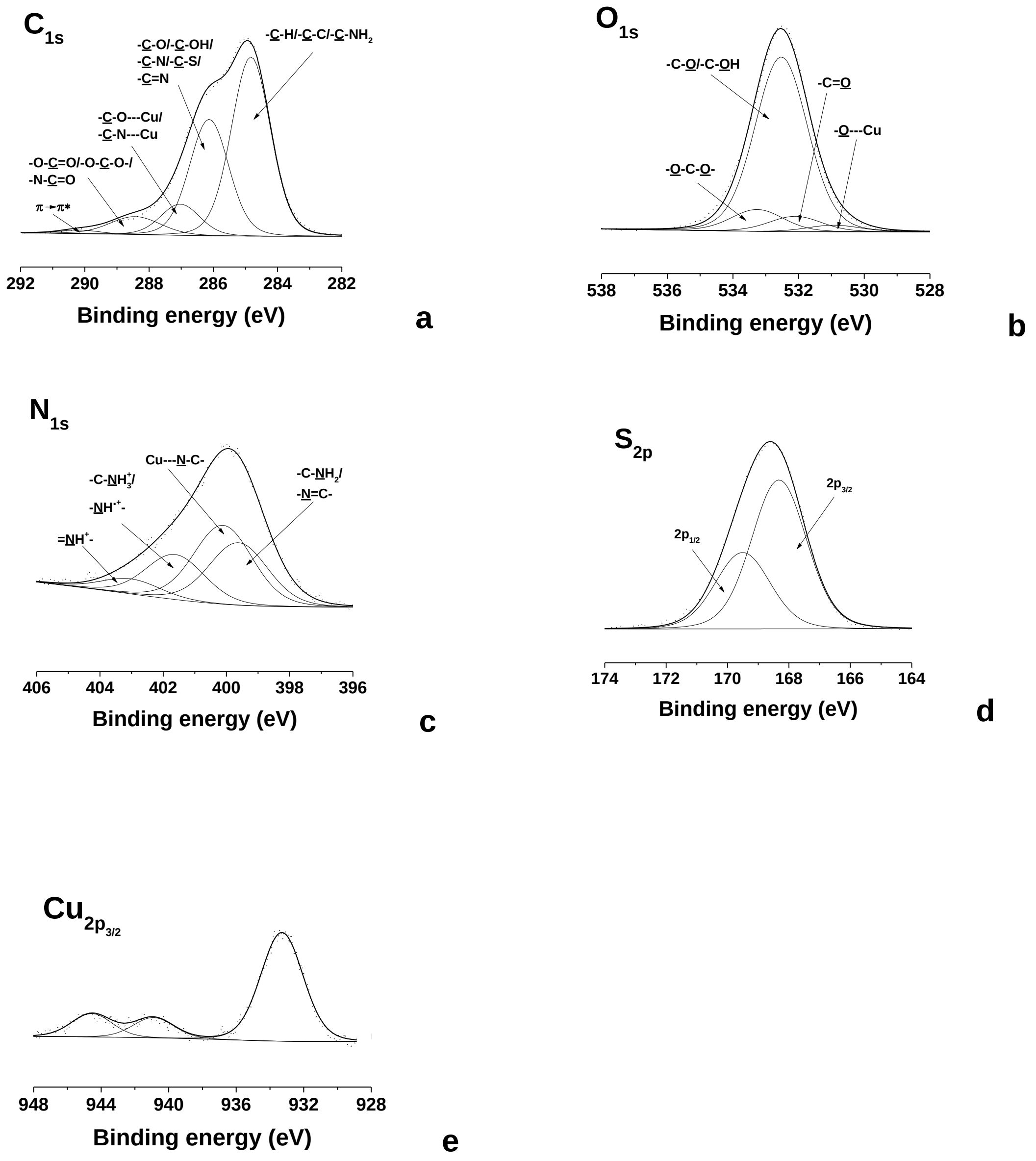


**Figure S7**. XPS peak fittings for $Cu^{2+}$ complex of cr(PVA/Ch)/SQ (10 wt% SQ) mat [$C_{1s}$ (a), $O_{1s}$ (b), $N_{1s}$ (c), $S_{2p}$ (d), $Cu_{2p3/2}$ (e)].

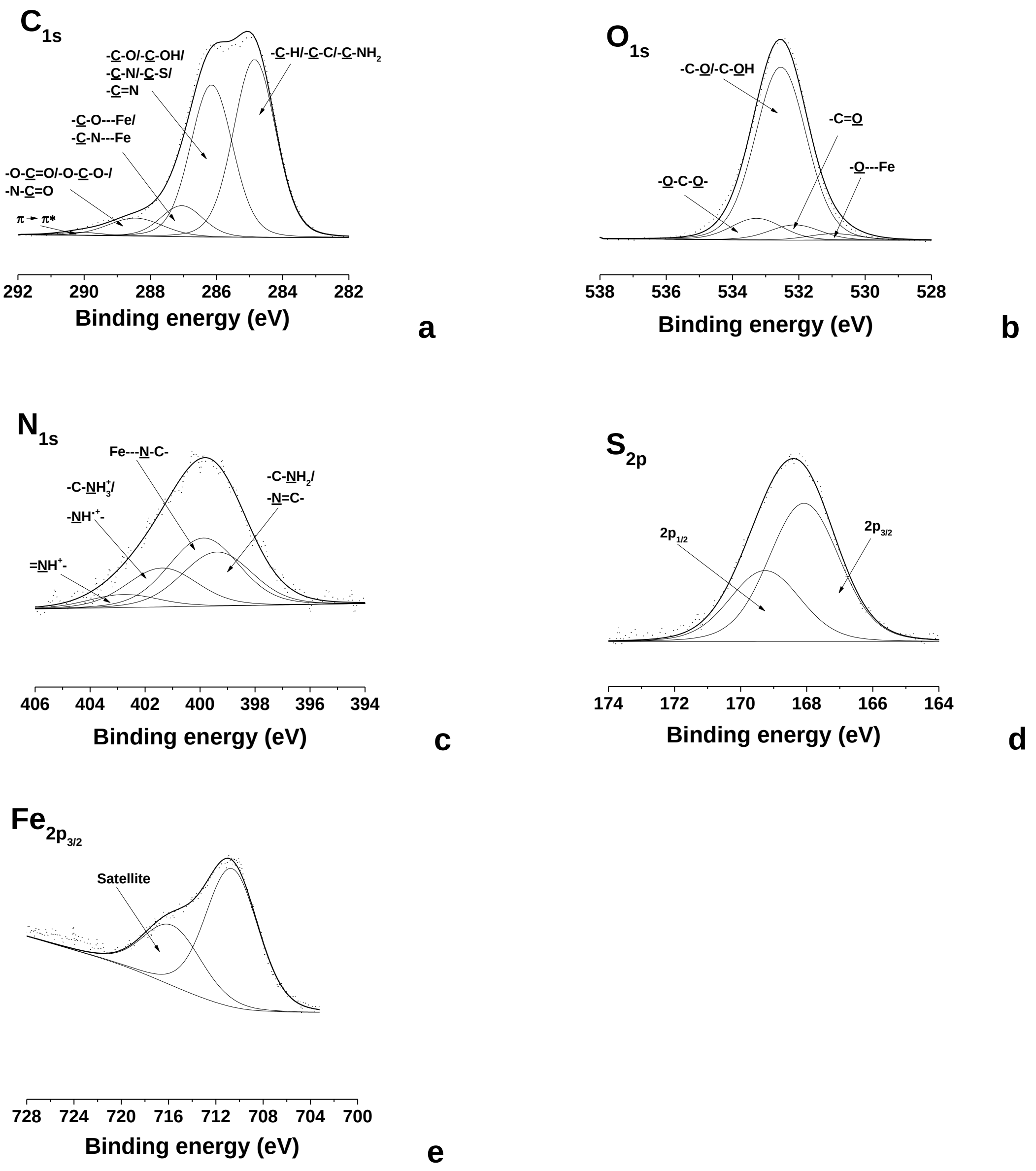


**Figure S8**. XPS peak fittings for $Fe^{3+}$ complex of cr(PVA/Ch)/SQ (10wt% SQ) mat [$C_{1s}$ (a), $O_{1s}$ (b), $N_{1s}$ (c), $S_{2p}$ (d), $Fe_{2p3/2}$ (e)].

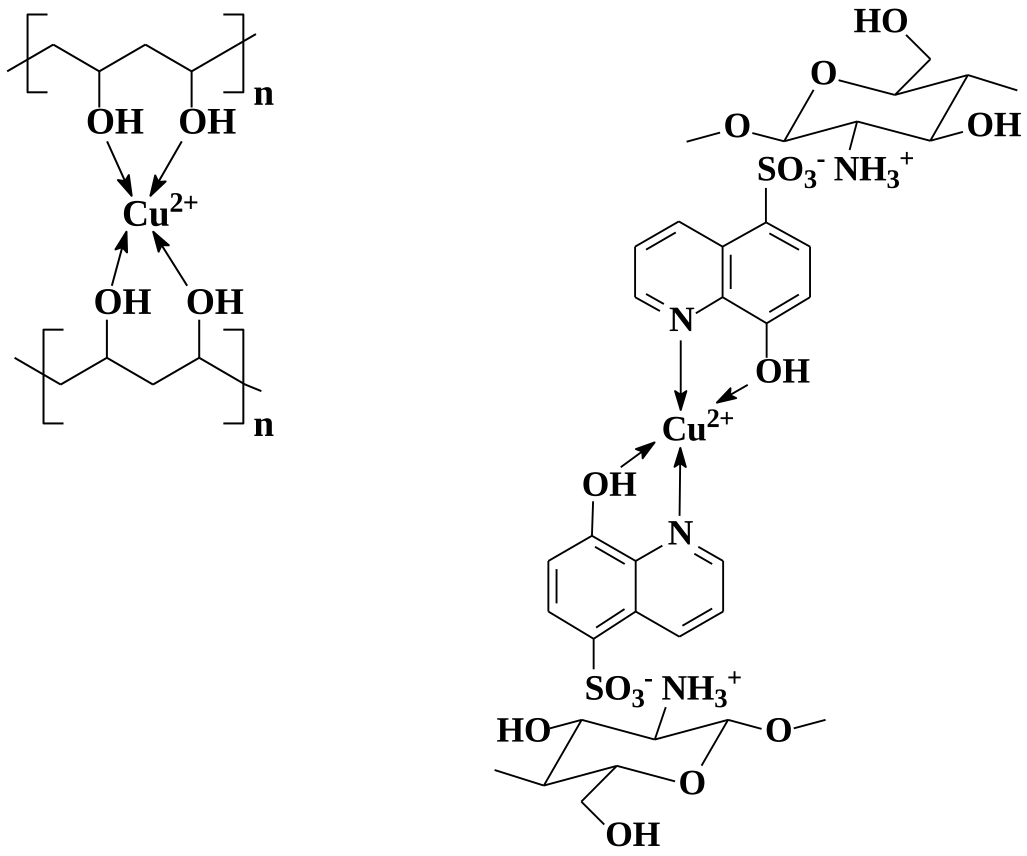


**a** **b**

**Scheme S1**. Schematic representation of: coordination of $Cu^{2+}$ with oxygen atoms of crPVA mat or of PVA incorporated in cr(PVA/Ch)/SQ mat (a) and coordination of $Cu^{2+}$ with oxygen and nitrogen atoms of SQ incorporated in cr(PVA/Ch)/SQ mat (b).

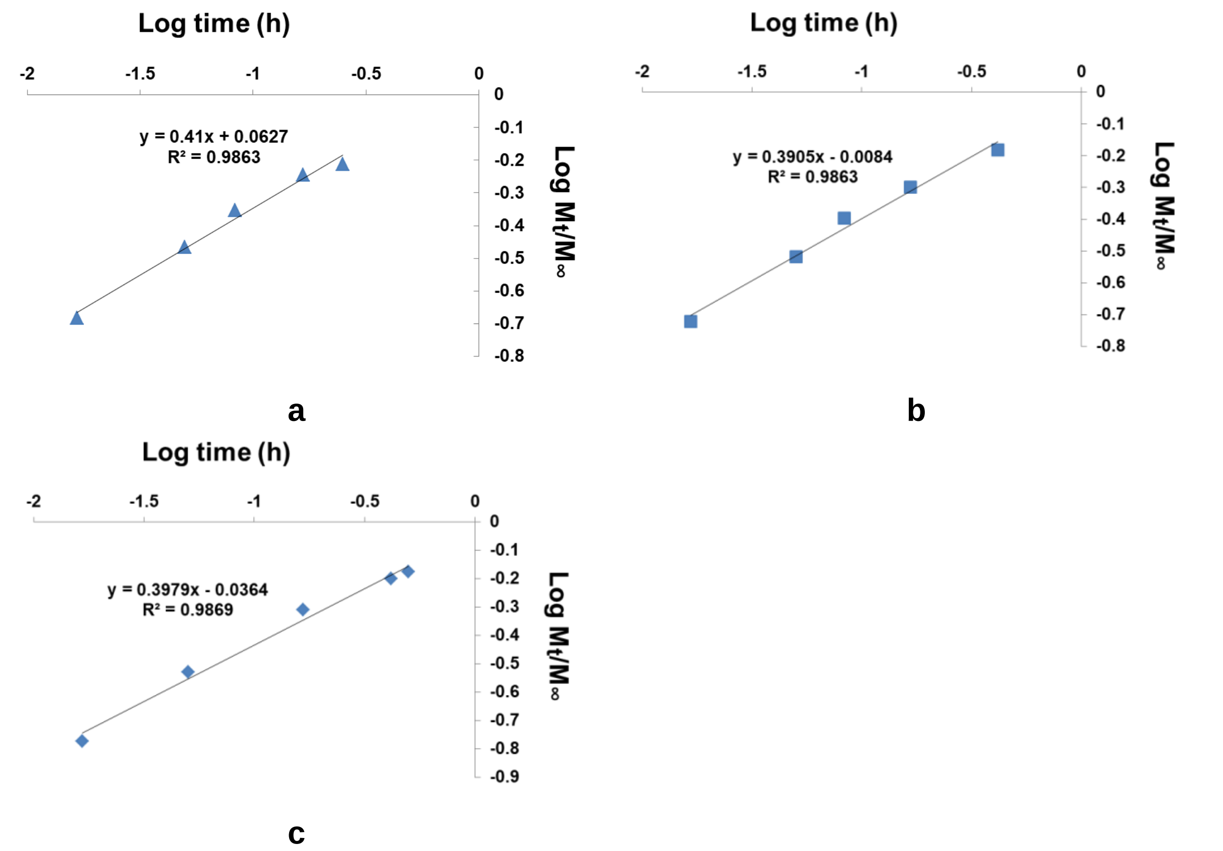


**Figure S9**. Korsmeyer-Peppas model of SQ, SQ.$Cu^{2+}$ and SQ.$Fe^{3+}$ release from: cr(PVA/Ch)/SQ (10 wt% SQ) mat (▲, a), $Cu^{2+}$ complex of cr(PVA/Ch)/SQ (10 wt% SQ) mat (■, b) and $Fe^{3+}$ complex of cr(PVA/Ch)/SQ (10 wt% SQ) mat (♦, c) in PBS/Tween 40 (99/1 v/v) at 37°C, pH 7.4, ionic strength 0.1.

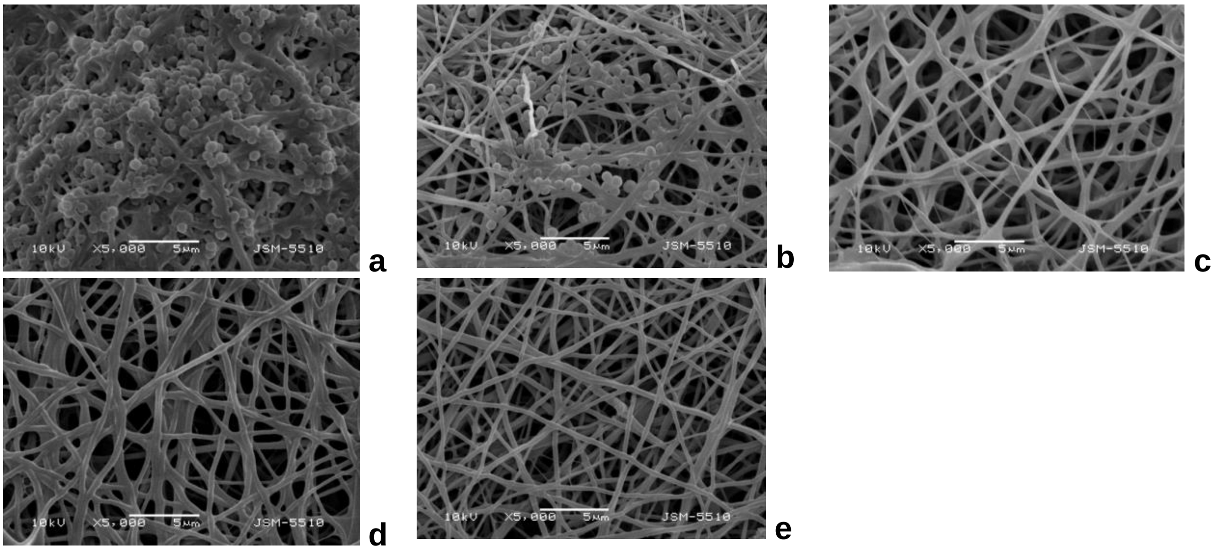


**Figure S10**. SEM micrographs of mats that have been incubated in *S. aureus* cell culture ($10^7$ cells/mL) for 24 h at 37°C: crPVA mat (a), cr(PVA/Ch) mat (b), cr(PVA/Ch)/SQ mat (c), $Cu^{2+}$ complex of cr(PVA/Ch)/SQ mat (d) and $Fe^{3+}$ complex of cr(PVA/Ch)/SQ mat (e); Magnification: 5000×.

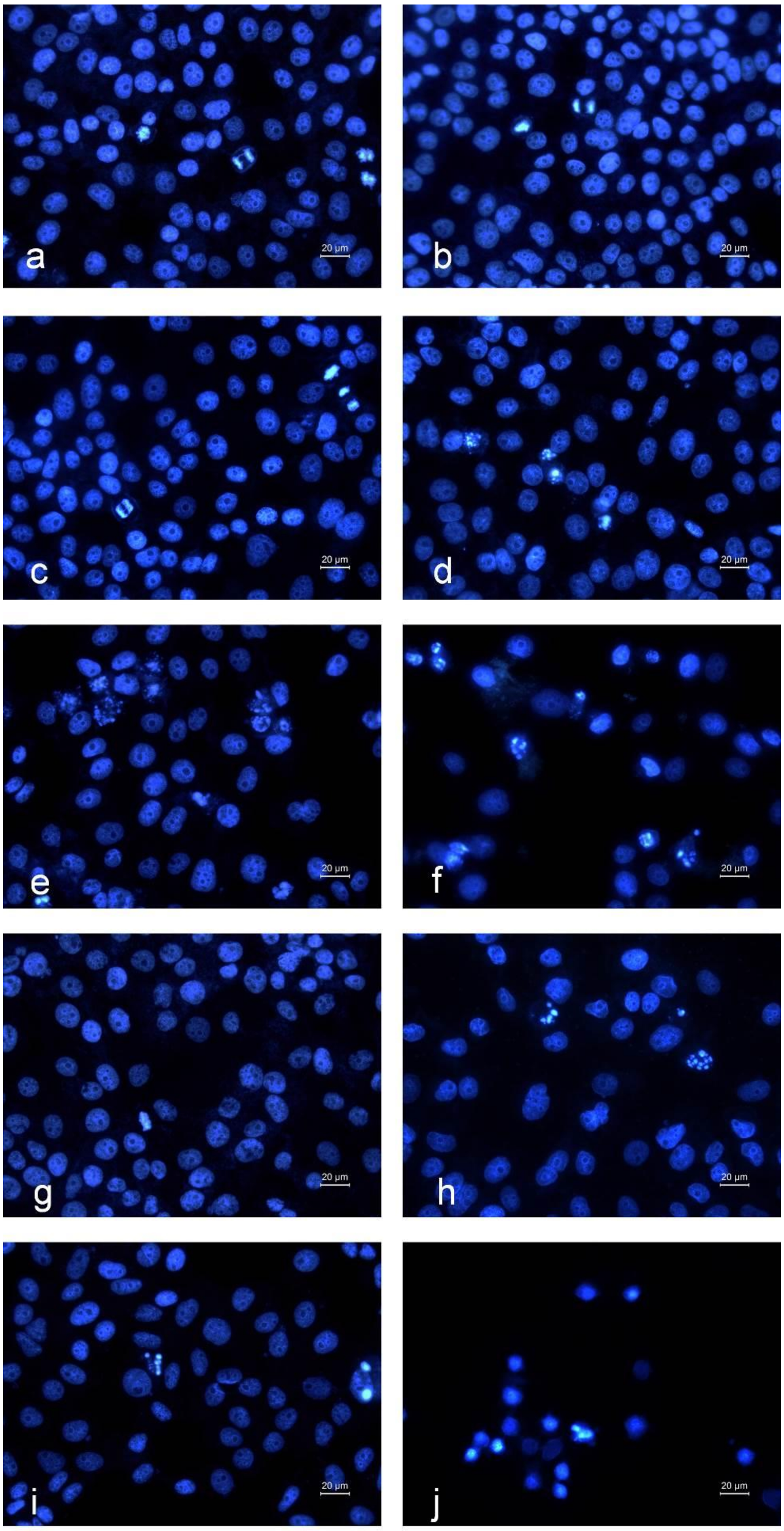


**Figure S11**. Fluorescence microscopic images of HeLa tumor cells stained with DAPI: (a) untreated HeLa cells (a control), (b) crPVA mat, (c) cr(PVA/Ch) mat, (d) cr(PVA/Ch)/SQ mat, (e) $Fe^{3+}$ complex of cr(PVA/Ch)/SQ mat, (f) $Cu^{2+}$ complex of cr(PVA/Ch)/SQ mat, (g) aqueous solution of Ch, (h) aqueous solution of SQ, (i) solution of SQ.$Fe^{3+}$, and (j) solution of SQ.$Cu^{2+}$; scale bar = 20 $\mu$m. All SQ- containing formulations and their $Fe^{3+}$($Cu^{2+}$) complexes were tested at a concentration of SQ 340 μg/mL of culture medium.

## References

1. Gubendran, A.; Kesavan, M.P.; Ayyanaar, S.; Raja, J.D.; Athappan, P.; Rajesh, J. Synthesis and characterization of water-soluble copper(II), cobalt(II) and zinc(II) complexes derived from 8-hydroxyquinoline-5-sulfonic acid: DNA binding and cleavage studies. *Appl. Organometal. Chem.* **2017**, e3708.
2. Jiang, F.; Xue, W. Preparation of tris(8-hydroxyquinoline-5-sulfonic acid) iron(III) complex and photocatalytic property of surface modified $TiO_2$. *Adv. Mat. Res.* **2012**, *399-401*, 1481-1486.
3. Sureshkumar, B.; Sheena Mary, Y.; Yohannan Panicker, C.; K.S. Resmi, S. Suma, Stevan Armakovic, Sanja J. Armakovic, C. Van Alsenoy, Spectroscopic analysis of 8-hydroxyquinoline-5-sulphonic acid and investigation of its reactive properties by DFT and molecular dynamics simulations. *J. Mol. Struct.* **2017**, *1150*, 540-552.
